# Exosomes mediate Coxsackievirus B3 transmission and expand the viral tropism

**DOI:** 10.1371/journal.ppat.1011090

**Published:** 2023-01-12

**Authors:** Yuxuan Fu, Sidong Xiong

**Affiliations:** Jiangsu Key Laboratory of Infection and Immunity, Institutes of Biology and Medical Sciences, Soochow University, Suzhou, China; Purdue University, UNITED STATES

## Abstract

Specific virus-receptor interactions are important determinants in viral host range, tropism and pathogenesis, influencing the location and initiation of primary infection as well as viral spread to other target organs/tissues in the postviremic phase. Coxsackieviruses of Group B (CVB) and its six serotypes (CVB1-6) specifically interact with two receptor proteins, coxsackievirus-adenovirus receptor (CAR) and decay-accelerating factor (DAF), and cause various lesions in most permissive tissues. However, our previous data and other studies revealed that virus receptor-negative cells or tissues can be infected with CVB type 3 (CVB3), which can also effectively replicate. To study this interesting finding, we explored the possibility that exosomes are involved in CVB3 tropism and that exosomes functionally enhance CVB3 transmission. We found that exosomes carried and delivered CVB3 virions, resulting in efficient infection in receptor-negative host cells. We also found that delivery of CVB3 virions attached to exosomes depended on the virus receptor CAR. Importantly, exosomes carrying CVB3 virions exhibited greater infection efficiency than free virions because they accessed various entry routes, overcoming restrictions to viral tropism. *In vivo* experiments demonstrated that inhibition of exosome coupling with virions attenuated CVB3-induced immunological system dysfunction and reduced mortality. Our study describes a new mechanism in which exosomes contribute to viral tropism, spread, and pathogenesis.

## Author summary

Enteroviruses of the family Picornaviridae, in particular coxsackieviruses of Group B (CVB), are highly prevalent human pathogens associated with a variety of acute and chronic forms of diseases. The presence of receptors on specific host cells and abundance of these cells in tissues are major determinants of the route of viral entry into a host. However, our previous data and other studies revealed that virus receptor-negative cells or tissues can be infected with CVB3, which can also effectively replicate. To study this interesting finding, we explored the possibility that exosomes are involved in CVB3 tropism and that exosomes functionally enhance CVB3 transmission. An increasing body of evidence has shown that exosomes released from virus-infected cells contain viral particles or genomes move to neighboring cells, contributing to virus dissemination. In our study, we found that exosomes were prevalent and robust vehicles for the delivery of CVB3 virions, resulting in effective infection of host cells. Additional data confirmed that delivery of CVB3 virions attached to exosomes surmounted barriers to viral tropism. Our novel findings provide new insight into how coxsackieviruses control and exploit exosomes for transmission in an infected host.

## Introduction

Virus-receptor interactions play key roles in viral host range, tissue tropism, and viral pathogenesis. Viruses leverage elegant mechanisms to attach to one or multiple receptors, overcoming the plasma membrane barrier to gain entry and subsequently access the cellular machinery that is required for their replication. Therefore, the presence of receptors on specific host cells and abundance of these cells in tissues are major determinants of the route of viral entry into a host.

Enteroviruses of the family Picornaviridae, in particular coxsackieviruses of Group B (CVB), are highly prevalent human pathogens associated with a variety of acute and chronic forms of diseases, such as viral myocarditis, aseptic meningitis, and pancreatitis in both infants and adults [1,2]. CVB and its six serotypes (CVB1-6) are nonenveloped icosahedral particles with a diameter of approximately 30 nm. The life cycle of CVB begins when the virus binds CAR (coxsackievirus and adenovirus receptor) to gain cell entry [3,4]. In addition, CVBs bind DAF (decay-accelerating factor), a coreceptor with CAR, to infect cells [5]. In fact, the level of tissue permissiveness to viral infection is determined by virus receptors of the host. However, recent data have shown that the differential abundance of CAR or DAF receptors in respective tissues did not highly correlate with susceptibility of CVB type 3 (CVB3) infection [4,6]. Indeed, splenic tissue, which does not express CAR, showed a high viral load during acute infection [7]. Importantly, immune cells in the spleen and lymph nodes, such as T or B lymphocytes, which contribute to the spread and dissemination of a virus within a host, are persistently infected by CVB3 in the absence of the CAR receptor [8–10]. In addition, the vascular endothelium is prone to infection with CVB3, where it effectively replicates [11,12]. However, the CAR receptor is lacking in the vascular endothelium [13]. Therefore, other anonymous determinants may play important roles in tissue permissiveness and disease severity during CVB3 infection.

Extracellular vesicles (EVs) constitute a heterogeneous group of cell-derived membranous structures comprising exosomes and microvesicles that originate from the endosomal system or are shed from the plasma membrane, respectively. These vesicles have emerged as important to cell-to-cell communication and transfer of biologically active proteins, nucleic acids and lipids [14]. An increasing body of evidence has shown that extracellular vesicles or exosomes released from virus-infected cells contain viral particles, genomes, or other pathogenic factors move to neighboring cells, contributing to virus dissemination and productive infection [15,16]. For instance, exosomes released from enterovirus 71-infected cells contained full-length viral RNA, which were successfully transferred to nonpermissive cells to generate new infectious viral particles [17]. Additionally, hepatitis A virus, previously considered to be a nonenveloped virus, has recently been shown to be secreted within exosomes that can potentially be transported to uninfected cells in an infected individual [18]. These observations indicated that the envelopment of intact virion or viral genomes by host membranes may expand the tropism of a virus, as exosome surface proteins engage target cells not viral surface proteins [15,19,20]. This may provide an additional route for the spread of a virus within initial target organs and systemic spread to distant organs. Therefore, it is likely that extracellular vesicles or exosomes may provide potential alternative CVB3 infection pathways that are not dependent on the expression of virus receptors.

In this study, we concentrated on EV participation during CVB3 infection and the functions of EVs that are enhanced during viral transmission. We found that exosomes were prevalent and robust vehicles for the delivery of CVB3 virions, resulting in surmounting infection inhibition caused by viral tropism by leveraging various entry pathways. Importantly, inhibition of exosome coupling with virions attenuated CVB3-induced immunological system dysfunction and myocarditis pathogenesis. Our findings offer a new perspective for understanding CVB tropism, spread, and pathogenesis.

## Results

### CVB3 effectively infected virus receptor-negative cells and tissues

**Fig 1:**
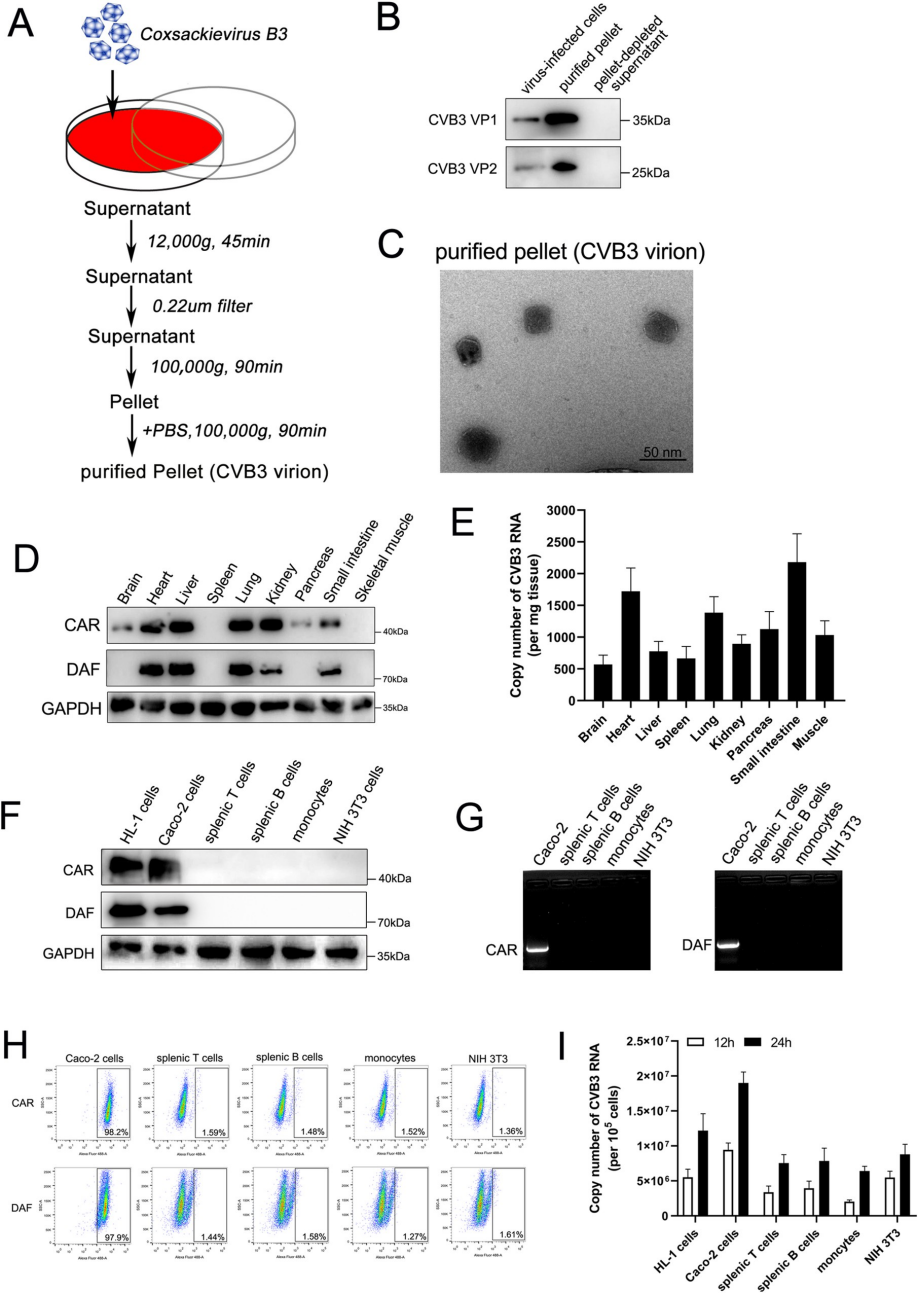
CVB3 effectively infected virus receptor-negative cells and tissues. (A and B) Caco-2 cells were infected with coxsackievirus B3 at 0.05 TCID_50_ for 24h. The purified pellet was isolated from culture supernatant by differential ultracentrifugation(A) and analyzed by western blot with CVB3 structural protein VP1 and VP2 specific-antibody. (C) The ultracentrifugal pellet containing CVB3 virion was analyzed by transmission electron microscope. Bar = 50nm. (D) Western blot analysis of the main receptor CAR and co-receptor DAF in various mouse organ tissues. (E) Real-time PCR data for the copy number of CVB3 RNA in different mouse organ tissues after infecting with CVB3 that isolation by differential ultracentrifugation. Six weeks-old of mice were orally inoculated after fasting at 5×10^6^ TCID_50_ per mouse for 24h. n = 5. (F, G and H) The expression levels of CAR and DAF in different primary immune cells or cell lines were determined by western blot analysis(F), RT-PCR(G) and flow cytometry(H). (I) Real-time PCR data for the copy number of CVB3 RNA in different primary immune cells or cell lines after incubating with ultracentrifugal CVB3 pellet. Data are shown as mean ± SD of three independent.

To characterize the infectivity of CVB3 in host cells, CVB3 virions were isolated from the supernatant of Caco-2-infected cell cultures via ultracentrifugation at 100,000×g (Fig 1A). Western blot analysis showed that the CVB3 virion was highly enriched in the purified pellet, as indicated by the high levels of the virus-structural proteins VP1 and VP2 (Fig 1B). Complete virions were also observed by transmission electron microscopy, and the typical virion diameter was thus measured to be ~30 nm (Fig 1C). These data demonstrated that this ultracentrifugal isolation method was sufficient for isolating CVB3 virions. Then, the abundance of the main receptor CAR and coreceptor DAF in different mouse tissues was analyzed by western blotting. As shown in Fig 1D, high protein expression levels of CAR and DAF receptors were found in many organs, such as the heart, liver, lung, kidney and small intestine. Notably, CAR protein abundance was lower in the brain and pancreas. Neither of these two receptors was detected in the spleen or skeletal muscle. Nonetheless, all tissue organs were positive for CVB3 RNA after oral inoculation, although the levels of viral RNA copies differed (Fig 1E). In particular, the spleen and skeletal muscle, which did not express virus receptors, exhibited high CVB3 infection efficiency (Fig 1E). Moreover, we detected the protein expression of CAR and DAF receptors by western blotting with different types of cells. HL-1 (cardiac muscle cells) and Caco-2 cells exhibited high expression levels of CAR and DAF, but these proteins were not detected in splenic T cells, splenic B cells, monocytes or NIH 3T3 cells (Fig 1F). Correspondingly, RT–PCR and flow cytometry analysis confirmed the lack of CAR and DAF in splenic T cells, splenic B cells, monocytes and NIH 3T3 cells (Fig 1G and 1H). Then, we found that all these cells exhibited not only susceptibility to CVB3 infection, as indicated by a high copy number of viral RNAs, but also increased viral RNA copies with time (Fig 1I), suggesting that CVB3 efficiently entered and replicated in receptor-negative cells. These results demonstrated that CVB3 effectively infects and replicates in receptor-negative cells and tissue organs.

### CVB3 entry into receptor-negative cells depended on exosomes

**Fig 2:**
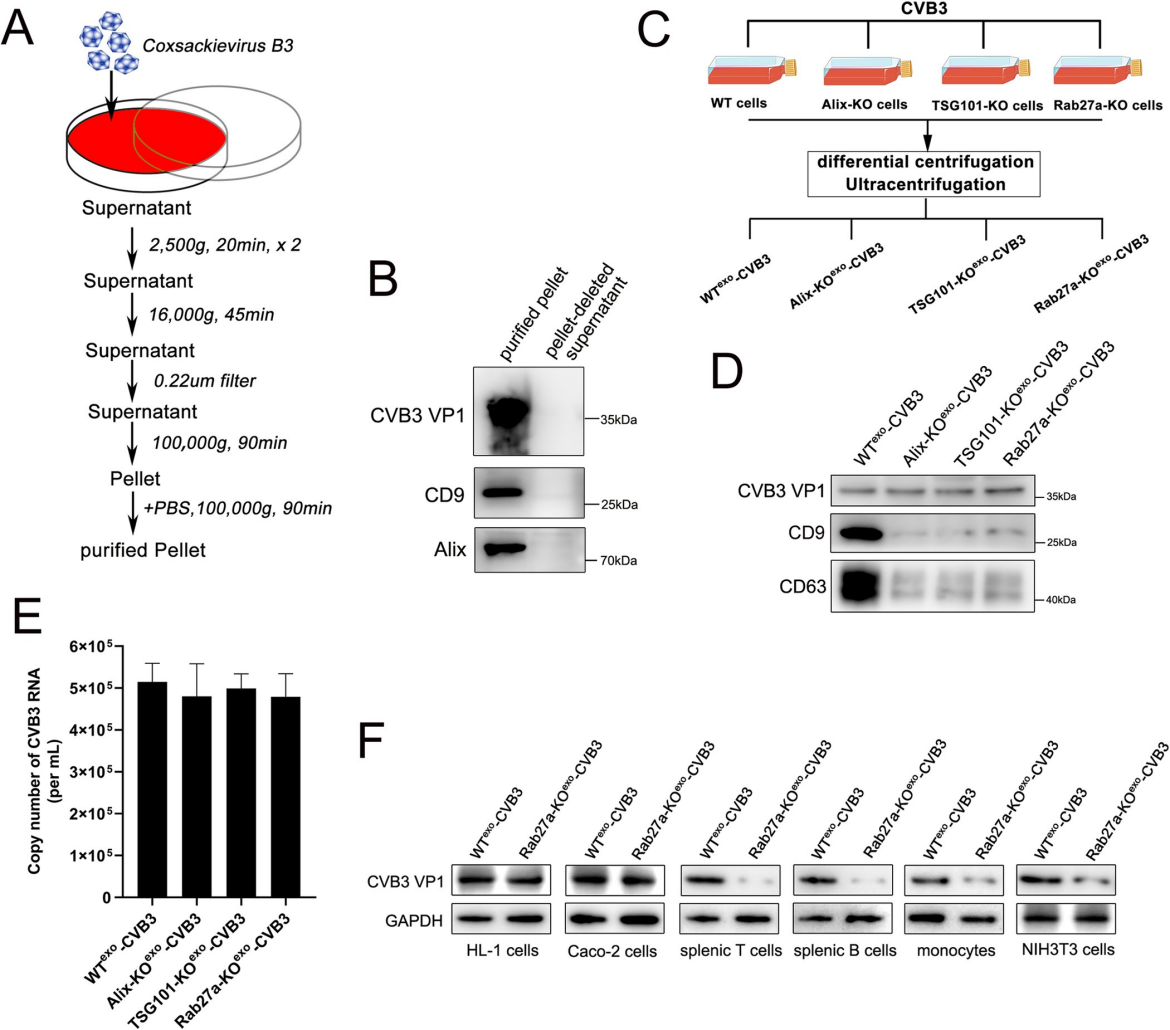
CVB3 entry into receptor-negative cells depended on exosomes. (A) Caco-2 cells were infected with coxsackievirus B3 at 0.05 TCID_50_ for 24h. The purified pellet containing exosomes and virion were isolated from culture supernatant by differential ultracentrifugation. (B) Western blot analysis of the purified pellet and pellet-deleted supernatant by CVB3 VP1 and exosomal markers CD9 and Alix. (C, D and E) The purified pellets were isolated from supernatant of CVB3-infected wild-type cells(WT cells) or knockout cells(KO cells). Equal number of WT or KO Caco-2 cells were infection with 0.05 TCID_50_ of CVB3 for 24h and the supernatants were collected, followed by differential ultracentrifugation(C). The protein levels of CVB3 VP1, exosomal markers CD9 and CD63 in different pellet were measured by western blot analysis(D). And the comparison of CVB3 RNA copy number by real-time PCR analysis(E). Data are shown as mean ± SD of three independent experiments. (F) Western blot analysis of viral protein VP1 in different type of cells after incubating with purified pellets that isolated from CVB3-infected WT cells or Rab27a-KO cells. Purified pellet containing exosomes and virion was isolated from equal numbers of infected WT cells or Rab27a-KO cells and then incubated each cell type for 12h.

Because enteroviral virions and exosomes are closely associated, the supernatant obtained after ultracentrifugation of CVB3-infected cells to isolate CVB3 virions may contain exosomes (Fig 2A). Indeed, after two rounds of ultracentrifugation at 100,000 × g, the CVB3 structural protein VP1 and exosome-related markers CD9 and Alix were found to be highly enriched in the pellet but not in the supernatant from which the pellet had been removed, as determined by western blotting (Fig 2B). To investigate the involvement of exosomes in CVB3 infection, we generated Alix, TSG101 or Rab27a genome knockout (GKO) Caco-2 cells, because these gene products have been reported to control exosome formation or secretion, using CRISPR/Cas9-mediated genome editing [14,21]. Wild-type (WT) or knockout (KO) cells were infected with CVB3 for 24 h, and then, viral particles and exosomes were isolated from the culture supernatant as described in Fig 2C. As shown in Figs 2D and 1E, we did not observe a difference in CVB3 VP1 or viral RNA levels between the WT (WT^exo^-CVB3) and KO cells (Alix-KO^exo^-CVB3, TSG101-KO^exo^-CVB3, Rab27a-KO^exo^-CVB3), indicating that the extracellular release of CVB3 virions was not affected by the inhibition of exosome secretion. In contrast, the levels of the exosome-related markers CD9 and CD63 in the supernatant of infected KO cells were significantly decreased compared with those in WT cell supernatant (Fig 2D). Then, we found that both HL-1 cells and HeLa cells exhibited highly efficient infection when treated with WT^exo^-CVB3 or Rab27a-KO^exo^-CVB3 for 12 h, as determined by western blotting with a specific anti-VP1 antibody (Fig 2F). However, the recipient splenic T and B cells, monocytes and NIH 3T3 cells, which lack virus receptors, showed inefficient infection after treatment with Rab27a-KO^exo^-CVB3 compared to that after infection with WT^exo^-CVB3 (Fig 2F). Together, our results suggested that exosome deficiency diminished the infectivity of CVB3 in host cells that lacked virus receptors.

### Exosomes as carrier vehicles for CVB3 virions

**Fig 3:**
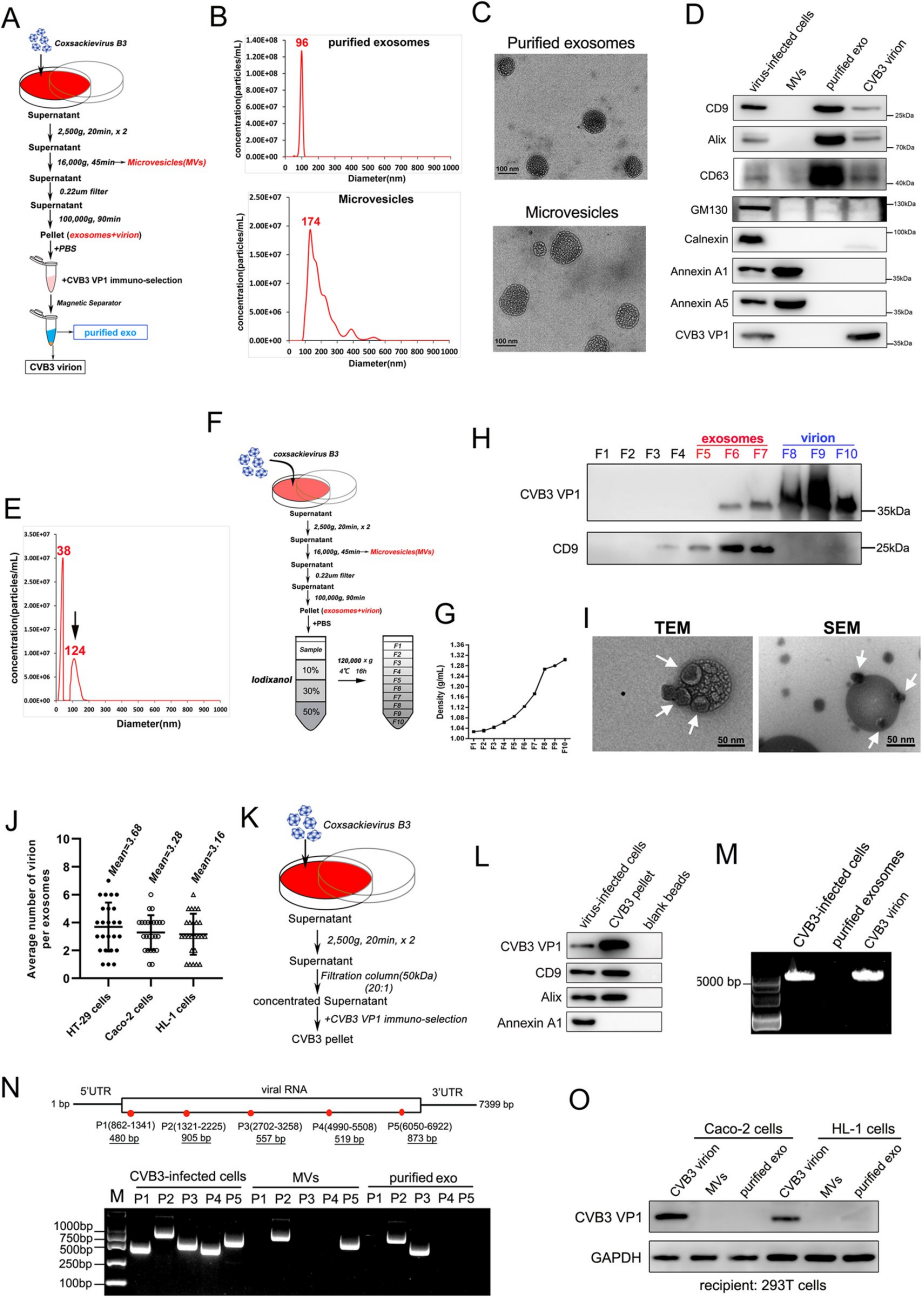
Exosomes as carrier vehicles for CVB3 virions. (A) Schematic presentation of immuno-magnetic isolation method to separate microvesicles(MVs) virion and exosomes from CVB3-infected Caco-2 cells. Caco-2 cells were infected with CVB3 at a 0.05 TCID_50_ for 1h, then the cells were washed and switched to medium supplemented with EV-depleted FBS for the production of MVs, exosomes and virus particles. (B and C) The purified exosomes and MVs were analyzed by Nanoparticle Tracking Analysis(NTA)(B) and transmission electron microscopy(C). (D) Purified exosomes, MVs and CVB3 virion derived from infected-cells above described were analysed by western blot with exosomal markers CD9, CD63 and Alix, non-exosomal markers GM130 (Golgi marker) and calnexin (ER marker), MVs markers Annexin A1 and A5. CVB3-infected cell lysates as positive control. (E) The CVB3 virion pellet isolated by immuno-magnetic method was were analyzed by NTA. The exosomes-CVB3 attachment section was indicated with the black arrow. (F, G and H) The iodixanol density gradient procedure to separate the pellet that from ultracentrifugal supernatants of CVB3-infected cells. A 10–50% Optiprep gradient was collected in 10 fractions(F1-F10) and analysed by western blot. CVB3 virion and exosomes was determined using specific anti-VP1 and anti-CD9, respectively(H). (I) Fractions 6–7 from Optiprep gradient purification were pooled, and transmission/scanning electron microscopy analysis of exosomes-CVB3 attachment. Viral particles were indicated with the white arrow. (J) Quantitation of CVB3 virion coupled with exosomes from images of electron microscopy in different cell lines. n = 25 exosomes from three independent experiments. Data are shown as mean ± SD. (K and L) The molecular weight cut off (MWCO) of ultrafiltration column(50kDa) was used to concentrate culture medium, followed by anti-VP1 beads or blank-beads immuno-selection. Then the CVB3 pellet and blank-beads pellet was determined by western blot analysis. (M and N) RT-PCR analysis of CVB3 genomic RNA in purified exosomes and MVs that isolated from CVB3-infected Caco-2 cells by differential ultracentrifugation according to description above. Total RNA extracted from CVB3-infected cells as positive control. (O) Western blot analysis of viral protein VP1 in recipient 293T cells. The 293T cells were incubated with CVB3 virion, purified exosomes or MVs that isolated from CVB3-infected Caco-2 or HL-1 cells for 24h.The whole cell lysates as determined by specific VP1 antibody. All data are presented as the mean± SD of three independent experiments.

To further explore whether the presence of exosomes affected CVB3 infectivity, we optimized the immunomagnetic isolation method to isolate virions from the exosome fraction. Briefly, after differential ultracentrifugation of culture medium, the precipitated pellet (containing virions and exosomes) was mixed with a specific antibody against CVB3 VP1, and then, immune-affinity beads were used to separate virions from the pellet (Fig 3A). Purified exosomes and microvesicles (MVs), other important extracellular vesicles, were analyzed via nanoparticle tracking analysis (NTA). The NTA showed that the predominant diameter size of the MVs was 174 nm, which was larger than that of exosomes, for which the average diameter was approximately 96 nm (Fig 3B). This extracellular vesicle characteristic was verified through transmission electron microscopy, which showed that the size of the MVs and exosomes was consistent with that obtained through NTA (Fig 3C). Our results demonstrated that purified exosomes and CVB3 virions were effectively separated through the use of an immunomagnetic method. Purified exosomes were further characterized for the presence of the exosomal markers CD9 and Alix, as well as the nonexosomal markers GM130 (a Golgi matrix marker) and calnexin (an endoplasmic reticulum marker) by western blotting (Fig 3D). The MV fraction was shown to be positive for Annexin A1 and A5 proteins but negative for protein markers of exosomes. Remarkably, CD9 and Alix were detected in the CVB3 virion fraction that had been separated through immunomagnetic selection. Moreover, in CVB3 virion fraction, a peak indicating a virion size of 124 nm, in addition to the main peak at 38 nm, which was consistent with typical size of the CVB3 particles as determined by NTA, suggested that partial exosomes may have precipitated with the virions (Fig 3E). To confirm this finding, we optimized the iodixanol density gradient ultracentrifugation procedure to purify exosomes isolated from the supernatants of CVB3-infected cells (Fig 3F). Equal volumes of fractions 1–10 (F1-F10) were used for western blotting to analyze the distribution of proteins in the virion fraction containing exosomes. We found that the exosomal marker CD9 was predominantly observed in F5-F7, while the structural protein VP1 of CVB3 was detected at the highest levels in F8-F10, indicating the presence of CVB3 virions in the fractions with the highest density (Fig 3G and 3H). Nevertheless, a portion of virions was found floating on an iodixanol cushion in the fractions containing exosomes (F6-F7), suggesting that these virions had been sedimented with exosomes. To verify that this fraction was composed of a mixture of exosomes and virions, electron microscopy and scanning electron microscopy were performed with the F6-F7 gradient fractions. As shown in Fig 3I, CVB3 virions were clearly attached to exosomes, as shown by TEM and SEM. The approximate estimation of the ratio of virions bound to exosomes was approximately 3:1 in the three types of cell lines examined (Fig 3J). To exclude the possibility that exosome-virion attachment was associated with ultracentrifugal extrusion, a molecular weight cut off (MWCO) of the ultrafiltration column (50 kDa) was used to concentrate the culture medium at a low speed (~4,000 ×g), followed by the VP1 identification via the immunoselection method (Fig 1K). Then, we observed the presence of the exosomal markers CD9 and Alix in the CVB3 pellet without detecting the MV marker Annexin A1 (Fig 3L). The pellet obtained from the immunobead incubation fraction alone (blank beads) did not include either CD9 or Alix protein, suggesting that the beads did not nonspecifically bind with exosomes. In addition, the purified exosomes isolated from CVB3-infected Caco-2 cells showed no detectable complete CVB3 RNA sequences as determined via RT–PCR (Fig 3M). Nevertheless, several fragments of viral RNA were identified both in the purified exosomes and MVs using different PCR primers of genomic sites (Fig 3N). We also confirmed that recipient 293T cells did not express CVB3 VP1 after incubation with purified exosomes or MVs isolated from CVB3-infected Caco-2 or HL-1 cells, with the fragments of viral RNA in purified exosomes or MVs indicating inefficient infection of recipient cells (Fig 3O).

### Characterization of exosome-CVB3 attachment

**Fig 4:**
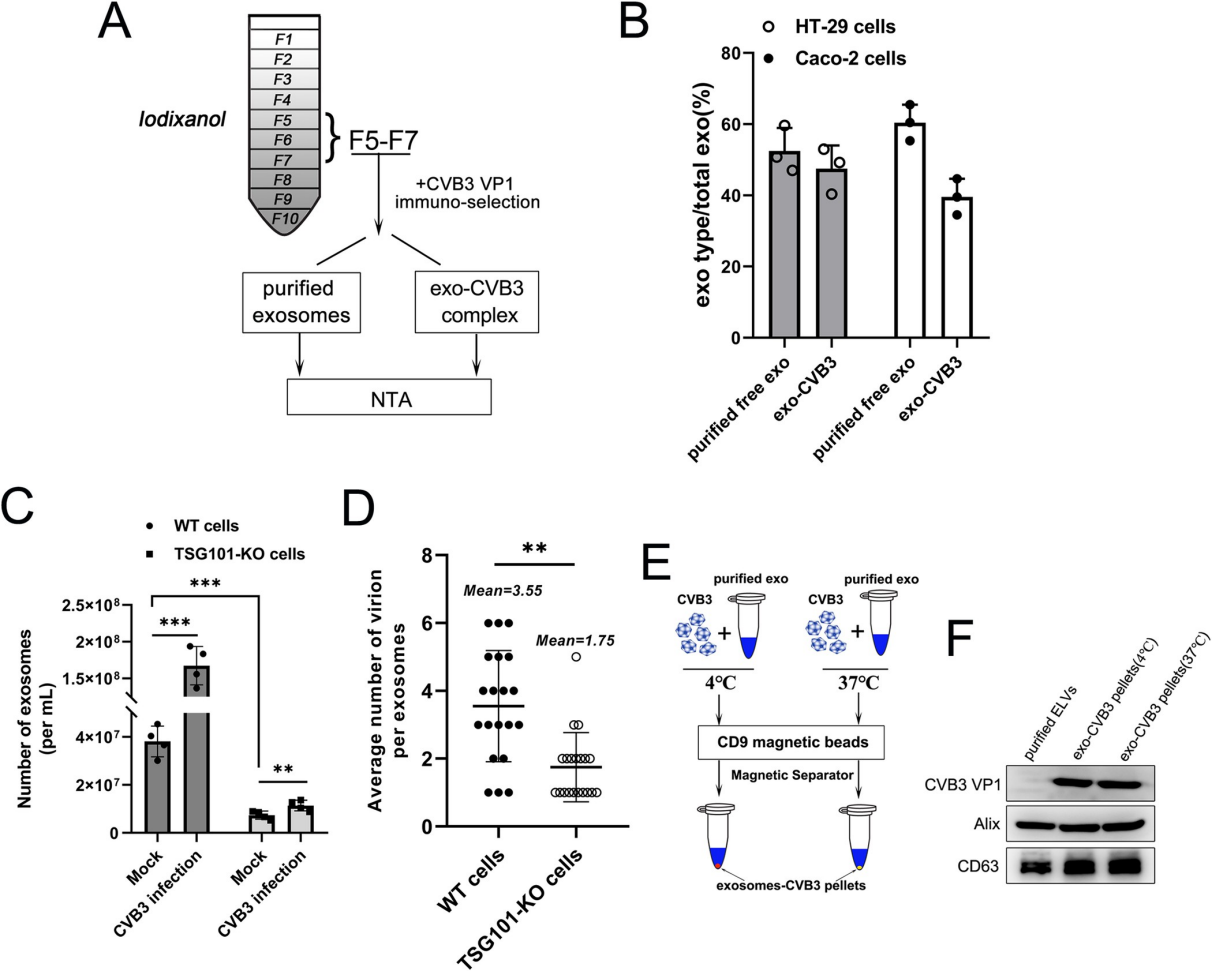
Characterization of exosome-CVB3 attachment. (A and B) The proportion of exosomes-CVB3 complex in total exosomes was determined by NTA. Fractions 5–7 from Optiprep gradient purification were pooled, and subjected to immuno-magnetic selection using VP1 antibody. Different symbols correspond to independent experiments. (C) The numbers of exosomes from WT Caco-2 cells or TSG101-KO Caco-2 cells with or without CVB3 infection was determined by NTA. Culture supernatants was collected from equal number of WT or KO Caco-2 cells at 24h post-infection with a 0.05 TCID_50_ of CVB3. (D) Quantitation of CVB3 virion bind with exosomes from images of electron microscopy in WT or KO Caco-2 cells. n = 20 exosomes from three independent experiments. (E and F) CVB3 virion mixed with purified exosomes at 4°C or 37°C temperature to form exosomes-virion complex followed by immuno-selection with anti-CD9. The exosomes-CVB3 pellets from different temperature condition were determined using viral anti-VP1 and anti-CD9 through western blot. Data are shown as mean ± SD of three independent experiments. (**p<0.01, ***p<0.001).

Next, we quantified the proportion of the exosome-CVB3 complexes in the total exosome population in the total supernatant of CVB3-infected cells with a known volume of exosomes. Exosomal fractions 5–7 (F5-F7) were subjected to immunomagnetic selection using the anti-VP1 antibody after iodixanol ultracentrifugation according to the aforementioned methods (Fig 4A). The total exosome volume was divided into two portions: (1) purified exosomes and (2) exosome-CVB3 complexes. We calculated 47% ± 5% and 39% ± 4% exosome-CVB3 complexes in the total exosome population isolated from CVB3-infected HT-29 and Caco-2 cells, respectively, as determined by NTA (Fig 4B). Moreover, we observed that CVB3 infected cells exhibited upregulated exosome production compared to mock-infected cells, but no obvious decrease in exosome secretion from CVB3-infected TSG101-KO cells was found (Fig 4C). We also found that the binding ratio of CVB3 to exosomes was significantly reduced in TSG101-KO cells (approximately 1.76 ± 0.9) compared to WT cells (approximately 3.47 ± 1.5) (Fig 4D). These results revealed that CVB3-induced exosome secretion resulted in increased virion attachment to exosomes. In addition, we mixed the CVB3 virion with purified exosomes at 4°C or 37°C to form an exosome-virion complexes and then subjected these complexes to immunoselection with an anti-CD9 antibody (Fig 4E). A Western blot analysis showed that CVB3 virions were still able to attach with exosomes at the lower temperature (Fig 4F), suggesting that virion-exosome binding was temperature-independent.

### Exosome coupling with CVB3 virions depended on CAR but not DAF

**Fig 5:**
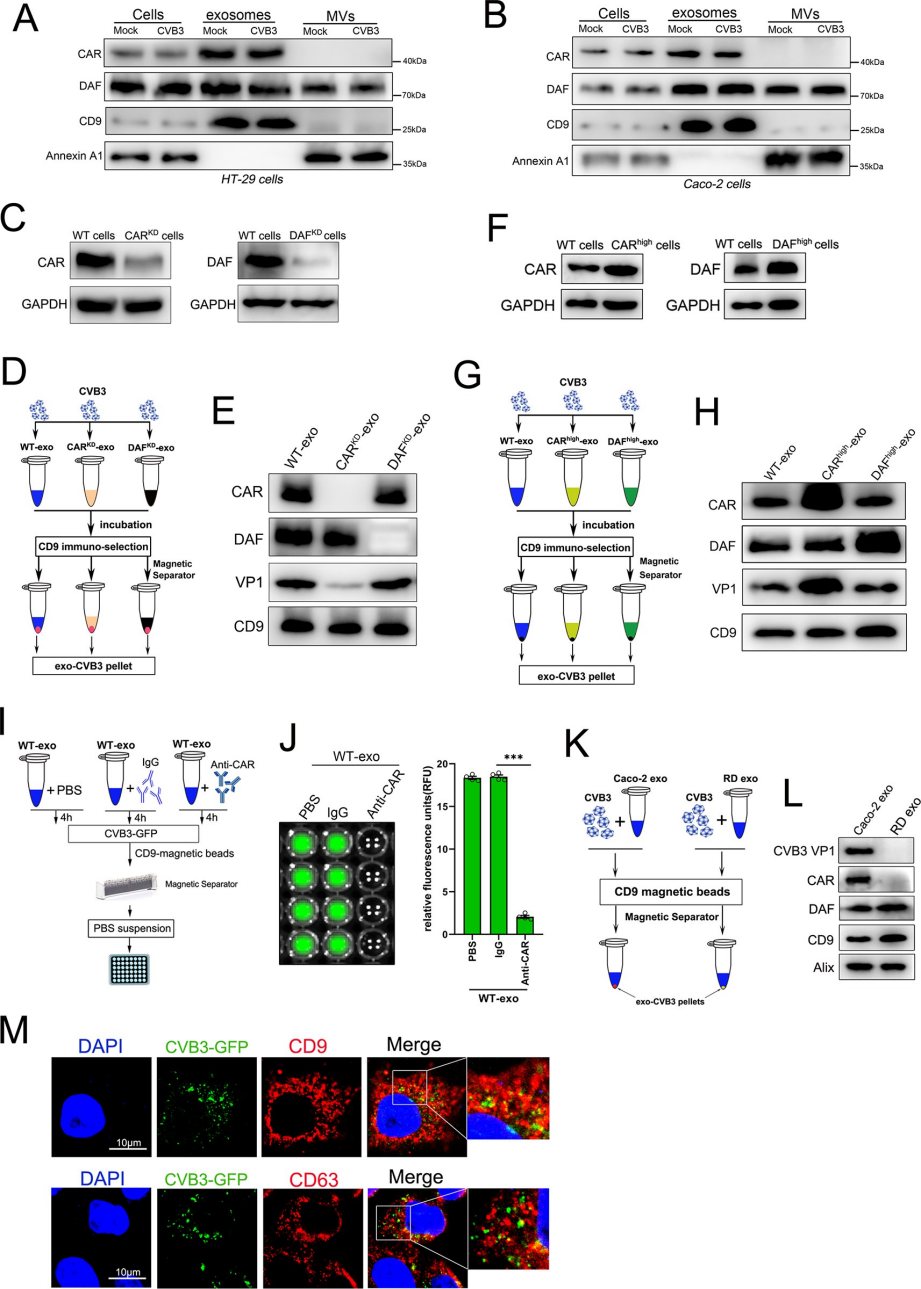
Exosome coupling with CVB3 virions depended on CAR but not DAF. (A and B) The exosomes and MVs derived from CVB3-infected cells or mock cells were analyzed by western blot with antibodies of viral receptors(CAR and DAF), exosomal markers(CD9) and MVs markers(Annexin A1). EVs were collected from equivalent amounts of culture medium, conditioned by equal numbers of cells, for equal lengths of time. The whole cell lysates as positive control. (C) Western blot analysis of CAR and DAF in stable knockdown Caco-2 cells(CAR^KD^ cells and DAF^KD^ cells). (D and E) Purified exosomes derived from WT cells, CAR^KD^ Caco-2 cells or DAF^KD^ Caco-2 cells were incubated with free CVB3 virion, followed by immuno-selection with anti-CD9(D). Then each group of the precipitated pellets were analysed by western blot (E). (F) Western blot analysis of CAR and DAF in stable overexpression Caco-2 cells(CAR^high^ cells and DAF^high^ cells). (G and H) Purified exosomes derived from WT cells, CAR^high^ Caco-2 cells or DAF^high^ Caco-2 cells were incubated with free CVB3 virion, followed by immuno-selection with anti-CD9 (D). Then each group of the precipitated pellets were analyzed by western blot (H). (I and J) Purified exosomes derived from WT Caco-2 cells were incubated with specific anti-CAR and then treated with CVB3-GFP virion, followed by CD9 immuno-selection. The PBS or IgG incubation as the negative control treatment(I). The relative fluorescence units (RFU) of each group was determined by Microplate Reader at 488nm of excitation wavelength (J). Data are shown as mean ± SD of three independent experiments. (***p<0.001). (K and L) Purified exosomes from HT-29 or RD cells were incubated with free CVB3 virion, followed by immuno-selection with anti-CD9(K). Then each group of the precipitated pellets were analysed by western blot (L). (M) Confocal co-localization analysis of CVB3 virion(green) and exosomal markers (CD9 and CD63, red) in Caco-2 cells. Caco-2 cells were infected with CVB3-GFP at 0.05 TCID_50_ for 24h and then incubated with anti-CD9 or anti-CD63 followed by incubated with fluorescent dye-tagged secondary antibody. Nuclei were stained with DAPI (blue). Bar = 10μm. All data are presented as the mean± SD of three independent experiments.

To explore the mechanisms involved in CVB3 attachment to exosomes, we considered whether the virus receptors contributed to this complex. We found both CAR and DAF receptors in the purified exosome fractions isolated from HT-29 cells and Caco-2 cells; however, only DAF receptors were detected in the MV fraction (Fig 5A and 5B). To determine whether either of these two receptors facilitate CVB3-exosome attachment, we constructed stable CAR- or DAF-knockdown HT-29 cells (CAR^KD^ cells or DAF^KD^ cells) and confirmed their successful establishment by western blotting (Fig 5C). Moreover, based on the immunological pull-down assay, purified exosomes were successfully isolated from WT cells (WT-exo), CAR^KD^ cells (CAR^KD^-exo) and DAF^KD^ cells (DAF^KD^-exo) and then mixed with CVB3 virions followed by subjection to immunoselection with an anti-CD9 antibody to exclude free virions from the results (Fig 5D). A Western blot analysis confirmed significantly lower CAR and DAF protein levels on the CAR^KD^-exo and DAF^KD^-exo groups than in the WT-exo group (Fig 5E). Moreover, we observed that the CAR^KD^-exo showed a significant reduction in binding with CVB3 virions compared to WT-exo and DAF^KD^-exo, as determined by an anti-CVB3 VP1 antibody. However, no difference in the quantity of bound CVB3 virions between WT-exo and DAF^KD^-exo was identified, suggesting that exosome-CVB3 attachment required the protein expression of CAR but not that of DAF on exosomes (Fig 5E). Furthermore, we established CAR or DAF overexpression in HT-29 cells (CAR^high^ cells or DAF^high^ cells) and isolated purified exosomes from CAR^high^ cells (CAR^high^-exo) and DAF^high^ cells (DAF^high^-exo) (Fig 5F). After mixing with CVB3 virions followed by immuno-selection with the anti CD9 antibody, the CAR^high^-exo exhibited a higher quantity of attached CVB3 virions than WT-exo or DAF^high^-exo (Fig 5G and 5H). To confirm the crucial role played by the CAR receptor in exosome-virion attachment, we carried out a blocking experiment by mixing exosomes with a specific anti-CAR antibody and then incubated the CVB3-GFP virions (generated from recombinant CVB3 cDNA tagged with the green fluorescent protein (GFP) gene) followed by immunoselection with the anti-CD9 antibody (Fig 5I). Fluorescence intensity assay illustrated that the relative fluorescence units (RFU) of the anti-CAR treatment group was significantly lower than that of the PBS or IgG treatment group, indicating that eliminating CAR on the surface of exosomes resulted in the inhibition of exosome-CVB3 attachment (Fig 5J). Consistently, purified exosomes from a rhabdomyosarcoma cell line that is CAR negative but DAF positive RD cells (RD-exo), failed to bind with CVB3 virions, in contrast to the exosomes from CAR-positive Caco-2 cells (Caco-2 exo), as indicated by an immunological pull-down assay (Fig 5K and 5L). Together, our gain- and loss-of-function experiments strongly supported the supposition that the main CVB3 receptor CAR was critical for exosome-CVB3 attachment but that the coreceptor DAF was not required. In addition, we found that CVB3-GFP particles were not colocalized with the exosomal marker CD9 or CD63 inside cells, suggesting that CVB3 did not attach to exosomes before being secreted; that is, the virions associated with the vesicles in the extracellular space (Fig 5M).

To determine whether exosome-virion attachment depends on virus-specific receptors, which is a common pathogen entry mechanism, we performed LC–MS/MS to analyze exosome samples isolated from three different cell lines (Caco-2, A549 and THP-1 cells). The MS/MS spectra showed that multiple virus receptors, as indicated by a search with the UniProt Knowledgebase (UniProtKB), were identified in each exosome sample obtained from different cells (S S1A, S1B and S1C Fig). To confirm that virus receptors on exosomes are involved in exosome-virion attachment, we chose human coxsackievirus A16 (CVA16), a nonenveloped, single-strand positive-sense RNA virus that causes hand, foot and mouth disease [22], and Zika virus, an arthropod-borne virus in the genus *Flavivirus* that can cause severe congenital malformations [23] to perform further experiments. Both the two kinds of virus were confirmed to have definite receptors and have similar diameter to CVB3 mature virion(~30nm for CVA16 and ~50nm for Zika). After differential ultracentrifugation of virus-infected culture medium or mock medium, the precipitated pellets (with virions and exosomes) were subjected to immunomagnetic selection with an anti-CD9 antibody to separate exosomes from the pellets (S1D Fig). Our results showed that the CVA16 structural protein VP1 and nonstructural protein 3AB were detected in the exosome fraction secreted from virus-infected Caco-2 cells, as determined by western blotting (S1E Fig). SCARB2 protein, the specific receptor of CVA16, was also observed in the exosome fraction, as indicated by the Alix and CD9 protein detection. Similarly, two structural proteins of Zika virus, the envelope and capsid proteins, were found in exosomes isolated from Zika-infected A549 cells. A receptor of Zika virus, Tyro3, was also detected in the exosome fraction (S1F Fig). These observations suggested that virions coupled with exosomes through specific virus receptors may be widespread during viral dissemination.

### Exosomes carrying CVB3 exhibited highly efficient infection of receptor-negative cells through various entry routes

**Fig 6:**
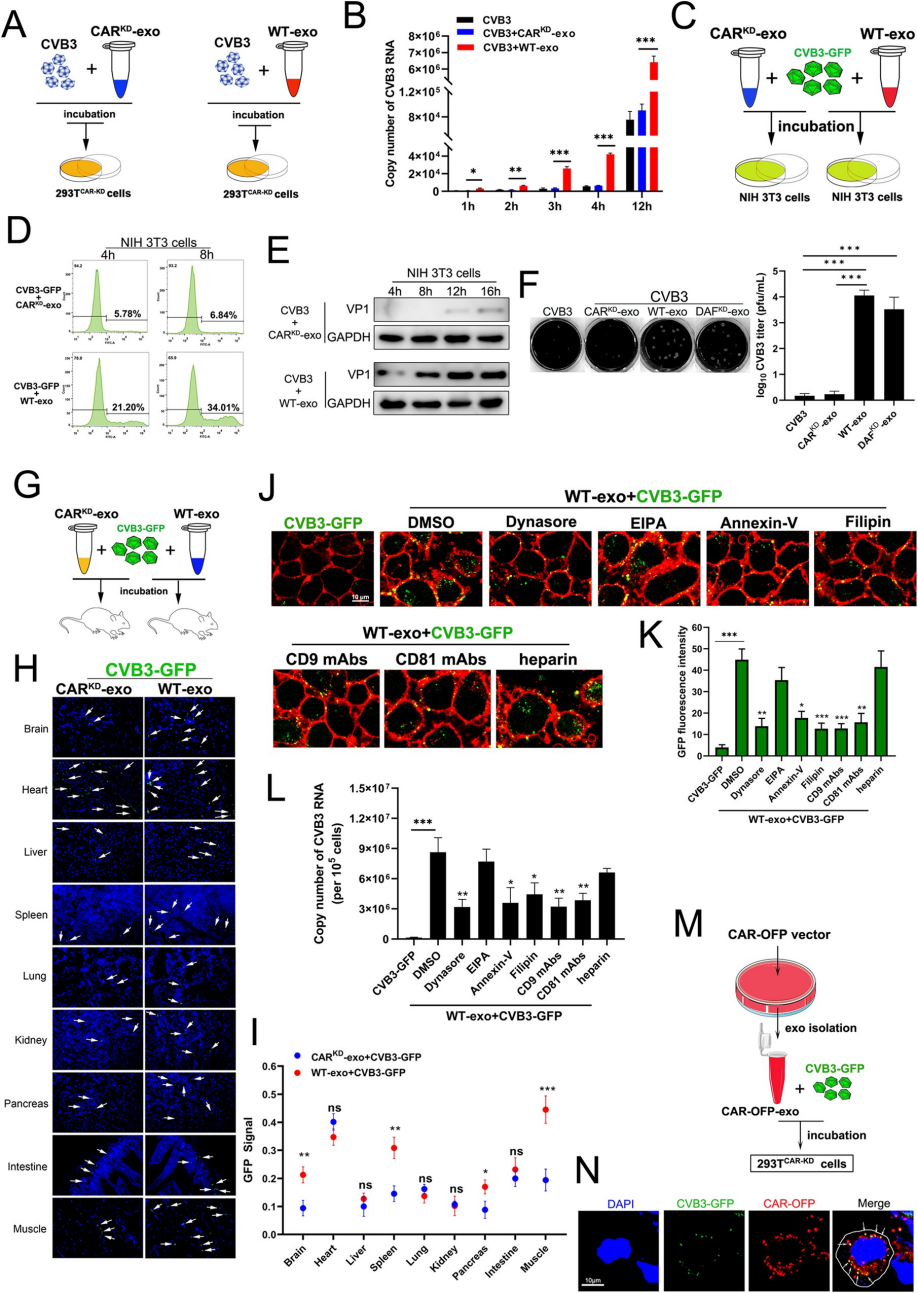
Exosomes carrying CVB3 exhibited highly efficient infection of receptor-negative cells through various endocytic pathways. (A and B) Purified exosomes derived from WT cells or CAR^KD^ Caco-2 cells were incubated with CVB3 virion for 4h. Then each group of exosomes-CVB3 complex was treated CAR-knockdown 293T cells(293T^CAR-KD^ cells) for different time-point(A). The copy number of viral RNA in 293T^CAR-KD^ cells was determined at various time points by real-time PCR analysis(B). Data are shown as mean±SD of three independent experiments. (C, D and E) Purified exosomes derived from WT cells or CAR^KD^ Caco-2 cells were incubated with CVB3-GFP virion, then each group of exosomes-CVB3-GFP complex was treated NIH 3T3 cells for different time-point(C). The condition of CVB3-infected cells was determined by flow cytometry analysis(D) and western blot(E). (F) Determination of virus titers by plaque assays. Each group of exosomes-CVB3 mixture was incubated monolayers of NIH 3T3 cells for 72 h, and then plaque assays were carried out to determine virus titers. Representative plaque formation on 3T3 cell monolayers were showed. (G, H and I) Equal copy number of CVB3-GFP virion mixed with CAR^KD^-exo or WT-exo and then injected into the tail veins of BALB/c mice(n = 5)(G). The tissue paraffin sections were examined at 24h post-injection for virion distribution(green) by immunofluorescence staining(H).The fluorescence intensity of GFP signal was examined through ImageJ software and a minimum of six different fields were measured(I). Data are shown as mean±SD of three independent experiments. (J and K) Confocal microscopy analysis of different routes for exosomes carrying CVB3 enter into recipient 293T^CAR-KD^ cells. Different drug or blocking-antibody were applied to 293T^CAR-KD^ cells followed by treating with free CVB3-GFP at 0.1 TCID_50_ or exosomes-CVB3-GFP complex(contain 0.1 TCID_50_ of CVB3-GFP) for 4h. DiI was used to label the cell membrane(Red)(J). The fluorescence intensity of accumulation GFP puncta was examined through ImageJ software and a minimum of six different fields cells were counted(K). Bar = 10μm. (L)Real-time PCR analysis of CVB3 RNA copy number in 293T^CAR-KD^ cells. After pretreating with drug inhibitors or blocking-antibodies, the cells were infected with free CVB3-GFP at 0.1 TCID_50_ or exosomes-CVB3-GFP complex(contain 0.1 TCID_50_ of CVB3-GFP) for 4h. (M and N) Purified exosomes from HeLa cells after transfecting with CAR-orange fluorescent fusion protein vector(CAR-OFP vector) mixed with CVB3-GFP virion, followed by incubating 293T^CAR-KD^ cells for 2h(M). Confocal co-localization analysis of CVB3 virion(green) and CAR receptor (red) in cells. Arrows point to CVB3-CAR colocalization(N). Bar = 10μm. All the data are shown as mean ± SD of three independent experiments. (*p<0.05, **p<0.01, ***p<0.001, ns: no significance).

To further explore the biological significance of exosomes carrying CVB3 virions, we constructed stable CAR-knockdown 293T cells (293T^CAR-KD^ cells) using the shRNA-lentivirus method. The CVB3 virions were mixed with CAR^KD^-exo or WT-exo for 2 h to form exosome-CVB3 complexes, and then add to 293T^CAR-KD^ cell cultures (Fig 6A). Real-time PCR showed that the copy number of viral RNA in 293T^CAR-KD^ cells was significantly higher after treatment with the CVB3-WT-exo complexes than after CVB3-CAR^KD^-exo or free CVB3 treatment at different time points (Fig 6B). Moreover, no significant difference in viral RNA levels in 293T^CAR-KD^ cells was observed between CVB3-CAR^KD^-exo and free CVB3 virion treatments (Fig 6B). In addition, CAR^KD^-exo or WT-exo were mixed with CVB3-GFP reporter virus and incubated with receptor-negative NIH 3T3 cells for various durations (Fig 6C). As shown in Fig 6D, CVB3-GFP-CAR^KD^-exo treatment led to a low rate of infection in recipient 3T3 cells at 4 h and 8 h; however, CVB3-GFP-WT-exo incubation resulted in a higher percentage of GFP-positive cells than CVB3-GFP-CAR^KD^-exo incubation at these time points (Fig 6D). Consistently, CVB3 VP1 was detected 4 h post-treatment with CVB3-GFP-WT-exos, in contrast to the time of detection after treatment with CVB3-GFP-CAR^KD^-exo, which was 16 h (Fig 6E), indicating that exosome-mediated viral entry was more efficient in receptor-negative cells. Together, our observations suggested that exosomes carrying virions facilitated CVB3 entry into receptor-negative cells, resulting in effective viral replication. Meanwhile, the effects of the exosomes on the yield of CVB3 progeny virus were also quantified by plaque assays. At 72 h post-infection, WT-exo and DAF^KD^-exo incubation resulted in a higher plaque count in NIH 3T3 cell monolayers compared with those of the CAR^KD^-exo or free CVB3 treatment, suggesting exosome-mediated CVB3 infection could enhance the production of infectious progeny virions(Fig 6F).

To further characterize exosome-mediated CVB3 infection entry *in vivo*, we mixed CVB3-GFP virions with CAR^KD^-exo or WT-exo and then injected them into mice. Mouse tissue sections were examined 24 h post-injection to detect virion distribution (Fig 6G). CVB3-GFP virions were significantly more enriched in the brain, spleen, pancreas and muscle of the mice injected with CVB3-GFP-WT-exos than in mice injected with CVB3-GFP-CAR^KD^-exo (Fig 6H and 6I), indicating that exosomes carrying CVB3 virions were taken up by mouse cells and entered tissue organs that lacked virus receptors with high efficiency *in vivo*. Cells appeared to take up exosomes through a variety of endocytic pathways [24]; therefore, to understand the routes through which exosome-carrying CVB3 virions enter cells, we treated cells with various inhibitory drugs that appeared to block specific exosome-uptake pathways. As shown in Fig 6J and 6K, the exosome-mediated entry of CVB3-GFP into recipient 293T cells treated with Dynasore, Annexin-V and Filipin, which have been confirmed to be inhibitors of clathrin/caveolin dependent, phagocytosis and lipid raft-mediated endocytic pathways, respectively, was significantly blocked. However, treatment of recipient cells with EIPA, an inhibitor of the micropinocytosis process, showed no effect on CVB3 virion entry. Nevertheless, complete abrogation of virus entry was not achieved with any of the tested inhibitors, suggesting that exosomes mediated viral entry through more than one route. The tetraspanin family is highly abundant on the exosome surface and contributes to exosome uptake [25]. Prevention of CVB3-GFP internalization was observed after tetraspanin CD9 or CD81 activity was blocked by specific antibodies (Fig 6J bottom). In addition, previous studies showed that cell-derived exosomes leverage heparin sulfate proteoglycans (HSPGs) for internalization [26]. However, treatment of cells with heparin (a heparin sulfate mimetic) did not affect the entry of CVB3-GFP, suggesting that HSPGs on the cell surface did not appear to play a direct role in exosome-mediated virion entry (Fig 6J bottom and 6K). Moreover, a real-time PCR analysis of viral copy number showed that virus infectivity was significantly reduced upon inhibitory drug or specific antibody treatment compared to the effect of DMSO, the control treatment, in NIH 3T3 cells (Fig 6L), indicating that exosomes facilitated the infectivity of CVB3 in receptor-negative host cells through different entry routes. To determine whether exosomes were indeed involved in the internalization of CVB3 virions, we isolated exosomes from HeLa cells that expressed CAR-orange fluorescent fusion protein (CAR-OFP-exo), mixed them with CVB3-GFP virions, and then incubated the mixture with 293T^CAR-KD^ cells (Fig 6M). A confocal analysis showed that CVB3-GFP colocalized intracellularly with the CAR-orange fluorescence-labeled exosomes (Fig 6N). This evidence demonstrated that exosomes were internalized into recipient cells together with CVB3 virions. Taken together, our results demonstrated that exosome-mediated CVB3 entry occurs by different routes, including various endocytic pathways and tetraspanin-dependent pathways. In addition, to investigate the infectivity of CVB3 carried by exosomes in receptor-positive cells, CVB3-GFP virions were mixed with equal numbers of CAR^KD^-exo, DAF^KD^-exo, and WT-exo. Fluorescence microscopy and flow cytometry analysis revealed the formation of exosome-CVB3 complexes that interacted with receptor-positive 293T cells at different time points (S2A Fig). We discovered that the presence of WT-exo facilitated CVB3-GFP entry into the recipient 293T cells to a significantly higher level than the PBS control or CAR^KD^-exo treatment, as measured between one hour and three hours after treatment; however, no significant difference between various treatment groups was found 4 hours post-treatment (S2B and S2C Fig). These data demonstrated that the infectivity of CVB3 mediated by exosomes was more efficient in permissive cells during the initial infection stage.

### Inhibition of exosomes coupling with CVB3 attenuated the pathogenetic events *in vivo*

**Fig 7:**
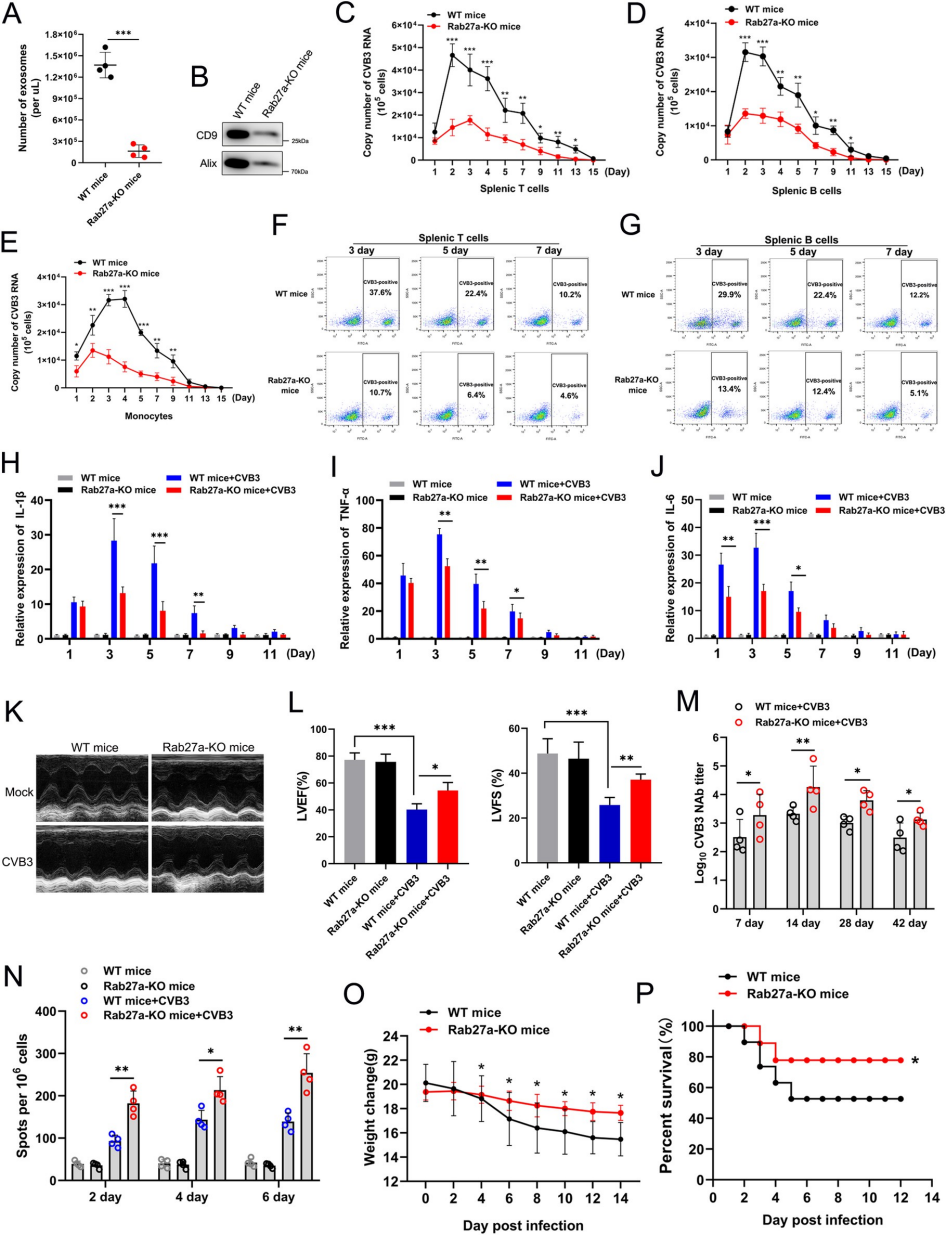
Inhibition of exosomes coupling with CVB3 attenuated the pathogenetic events *in vivo*. (A and B) The exosomes isolated from wild-type (WT) or Rab27a-KO mice of serum was quantified using NTA(A) and western blot(B). Each group contained 5 mice. (C, D and E) Real-time PCR data for the comparison of CVB3 RNA expression level in splenic T(C), splenic B(D) and monocytes(E) cells in WT or Rab27a-KO mouse at various time points. Six weeks-old of Rab27a-KO mice or WT mice were orally inoculated after fasting at 5×10^6^ TCID_50_ per mouse. n = 5 biologically independent animals per group. (F and G) The percentage of CVB3-infected splenic T(F) and splenic B(G) cells were determined by flow cytometric analysis at indicative time points. Cells were stained with anti-CVB3 and Alexa Fluor 488-conjugated anti-mouse IgG antibody. (H, I and J) Real-time PCR analysis of inflammatory factors expression in cardiac tissue of CVB3-infected mice or mock mice at various time points. (K and L) Left ventricular ejection fraction(LVEF) and left ventricular fractional shortening(LVFS) of CVB3-infected mice was measured though M-mode echocardiography. Rab27a-KO mice or WT mice(n = 5) were orally inoculated at 5×10^6^ TCID_50_ per mouse and the echocardiographic data was recorded at 3 day post-infection. (M) Levels of serum neutralizing antibody(NAb) titer in CVB3-infected mice. Different symbols correspond to independent experiments. (N) Cellular immune responses of CVB3-infected WT or Rab27a-KO mice was determined by IFN-γ ELISpot assay at 2, 4 and 6 day post-infection. (O and P) The body weight change (O) and survival rate (P) were respectively monitored daily until 14 day post-infection. All the data are shown as mean ± SD of three independent experiments. (*p<0.05, **p<0.01,***p<0.001).

To explore the role played by exosomes carrying CVB3 virions in pathogenicity *in vivo*, we constructed Rab27a-knockout mice (Rab27a-KO mice), because Rab27a is as a key regulatory gene product that controls exosome secretion. The number of serum exosomes in Rab27a-KO mice was significantly decreased compared to that in WT mice, as determined by NTA and western blotting (Fig 7A and 7B). Real-time PCR results showed a higher copy number of viral RNA in splenic T cells, B cells and monocytes from WT mice than in those from Rab27a-KO mice in a period of 1–11 days (Fig 7C, 7D and 7E). Correspondingly, splenic T cells in WT mice showed a higher rate of CVB3 infection than T cells in Rab27a-KO mice, as indicated by flow cytometry analysis on different days (Fig 7F). Similar results were also confirmed with splenic B cells from WT mice and Rab27a-KO mice (Fig 7G). These results demonstrated that a reduction in the number of exosomes diminished the efficiency of CVB3 infection of T and B lymphocytes, as well as monocytes. To investigate the pathogenetic events after inhibition of exosomes coupling with CVB3, we assessed the expression of IL-1β, TNF-α and IL-6 in the cardiac tissue of CVB3-infected mice and mock-infected mice. All inflammatory cytokines showed higher levels in the cardiac tissue of WT mice than in Rab27a-KO mice in the early stages of infection (within 7 days) (Fig 7H, 7I and 7J). Moreover, M-mode echocardiography revealed that CVB3-infected Rab27a-KO mice exhibited milder myocardial dysfunction than infected WT mice, as reflected by the left ventricular ejection fraction (LVEF) and left ventricular fractional shortening (LVFS) measurements (Fig 7K and 7L). These results suggested that inhibition of exosome secretion may relieve the CVB3-induced myocardial inflammatory response and mitigate myocardial dysfunction. To explore the role played by exosomes in CVB3-induced immune system loss, a CVB3-specific neutralizing antibody (NAb) and cellular immune responses were detected on different days. The NAb titers in CVB3-infected Rab27a-KO mice were higher than those in infected WT mice from 7 days to 42 days post-infection (Fig 7M). In addition, we observed that cellular immune responses in CVB3-infected Rab27a-KO mice were significantly stronger than those in infected WT mice, as indicated by an IFN-γ ELISpot assay performed on different postinfection days (Fig 7N). Notably, compared with WT mice, CVB3-infected Rab27a-KO mice exhibited less body weight loss and a higher survival rate (~80%) (Fig 7O and 7P). Taken together, our findings confirmed that inhibition of exosome-virion complex formation attenuated CVB3-induced immunological system dysfunction, prevented myocarditis pathology, and reduced mortality.

## Discussion

**Fig 8:**
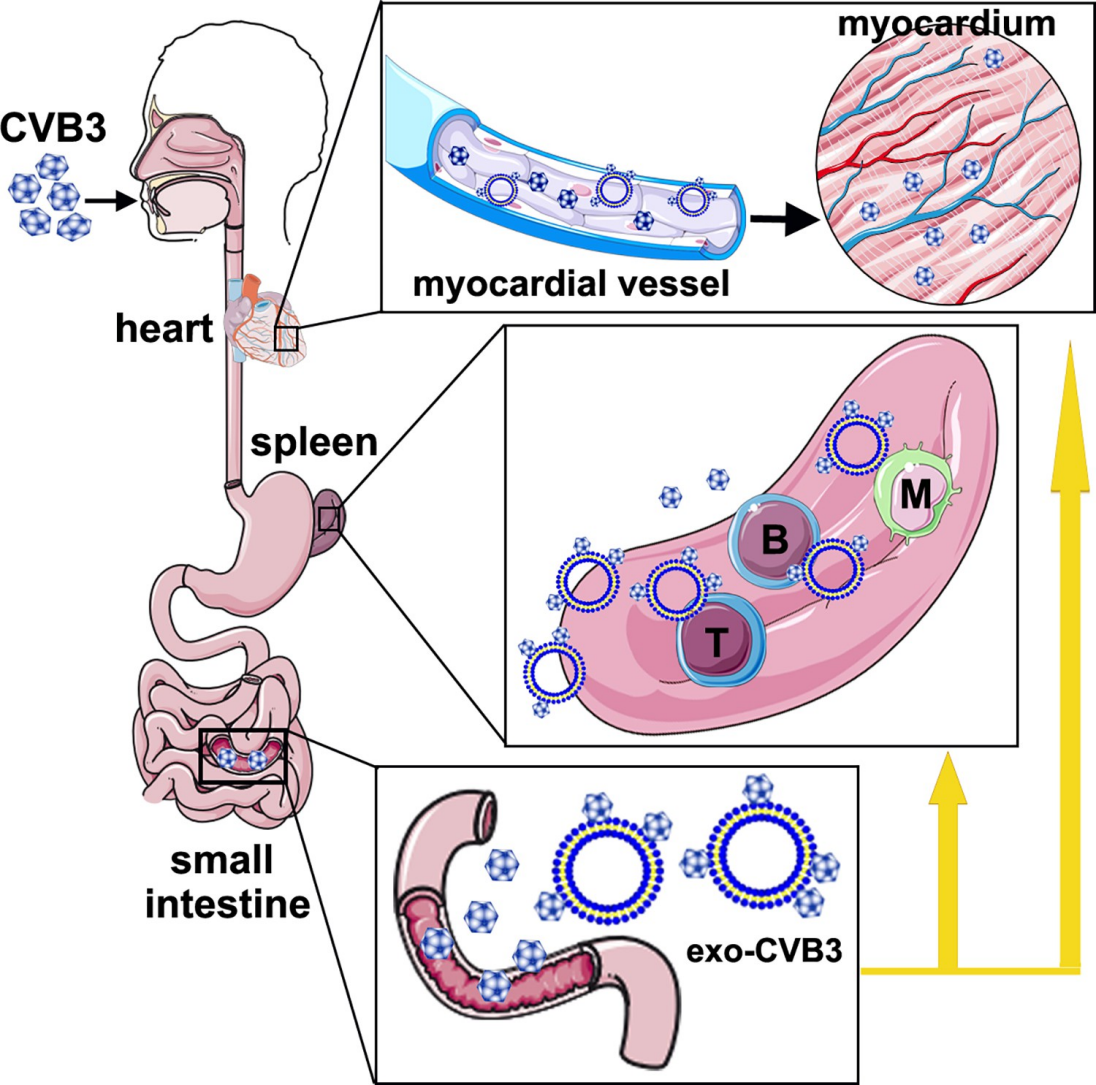
Exosomes provide a permissive route for CVB3 infection result in expanding viral tropism. CVB3 infects human through GI system and intestines are the entry portal of the viral infection. After enter in alimentary tract by the mouth, the virus initially replicates in intestine epithelial cells and subsequently spreads to the circulation and various organs. Meanwhile, exosomes also release from virus-infected epithelial cells into extracellular space. In this study, we find that exosomes are prevalent and robust vehicles for the delivery of CVB3 virions, resulting in entry of tissue organs such as spleen and myocardial vessel that lacked virus receptors with high efficiency. Persistent infection of lymphocytes provides CVB3 virus with an important way of disrupting the normal function of immune cells and the homeostasis-related immunological response to antigens, resulting in enteroviral myocarditis.

Extracellular vesicles (EVs) or exosomes are produced by virus-infected cells and are thought to be involved in intercellular communication between infected and uninfected cells. Studies confirmed that EVs are secreted from cells infected by viruses and that these EVs deliver viral particles, genomes, and other viral elements to neighboring cells, helping to establish productive infections and modulate the host immune response [15,27]. It is plausible that the envelopment of virions or the viral genome by EVs may expand the tropism of the virus, as EV surface proteins engage target cells not viral surface proteins. Importantly, this information may indicate an additional previously uncharacterized route of virus entry in a broader range of host cells than previously identified, because the evidence shows that viral infection is not dependent on the presence of virus-specific receptors. In this study, we found that exosomes were prevalent and robust vehicles for the delivery of CVB3 virions, resulting in effective infection of host cells. Additional data confirmed that delivery of CVB3 virions attached to exosomes surmounted barriers to viral tropism. Our novel findings provide new insight into how coxsackieviruses control and exploit exosomes for transmission in an infected host, particularly entry into receptor-negative cells such as lymphocytes (Fig 8).

The most common method used to isolate exosomes from cell culture supernatants involves a ultracentrifugation series. Notably, exosomes and many viruses share size, density, and molecular properties, therefore, it is imperative to separate exosomes and virions effectively to ensure that their respective functions are studied [27,28]. Our study optimized a two-step isolation approach by using an ultracentrifugation procedure combined with an immunomagnetic method to purify free CVB3 virions and purify exosomes from ultracentrifuged pellets. Using this protocol, we observed that an average of three complete CVB3 virions were coupled with one exosome. Inhibition of exosome secretion that had initially been induced by CVB3 significantly reduced the ratio of CVB3 virions bound to exosomes. Our data confirmed that CVB3 virions did not attach to exosomes before secretion and were thus extracellularly associated with vesicles. Importantly, the overall dispersion of virions needs to increase the cellular multiplicity of infection (MOI), which is defined as the average number of viral genomes that initiate the infection of a cell. Movement of virus aggregates or clumps give a virus a head start on cellular innate immune defenses [29,30]. Our study suggests that collective simultaneous viral infection is one way in which viruses enhance the MOI; that is, multiple virions are transported by each exosomes to infect other cells.

Viruses use cell surface receptors to initiate attachment to the plasma membrane and target specific cell types. Importantly, we found that both the CVB3-specific receptor and coreceptor, CAR and DAF, were present on the membrane surface of the exosomes. It is plausible that the presence of CAR or DAF on exosomes may contribute to the attachment of CVB3 to exosomes. In fact, our gain- and loss-of-function experiments showed strong evidence indicating that the main CVB3 receptor CAR was critical for exosome-CVB3 attachment but that the coreceptor DAF was not required. Notably, a previous study showed that soluble CAR bound to CVB3 with higher affinity than the equivalent form of DAF [31]. Interestingly, the interaction of CVB3 with the CAR receptor led to the formation of viral A-particles, which is common to all enteroviruses and characterized by partial exposure of viral RNA and release of the internal VP4 from the virus, but CVB3 interactions with soluble or cell surface DAF did not lead to viral A-particles [32,33]. Therefore, we speculated that by triggering conformational changes required to form A-particles by binding with the CAR on exosomes, CVB3 virions may undergo highly efficient replication after viral RNA is released into the cytoplasm. Extracellular vesicles have been classified as either exosomes or microvesicles (MVs) on the basis of their size and origin [34]. However, microvesicles isolated from virus-infected cells did not attach to CVB3 virions because of CAR receptor deficiency. Exosome biogenesis and release involve the endosomal sorting complex required for transport (ESCRT complex) and other associated proteins, such as Alix and TSG101. In contrast, microvesicles are produced directly through the outward budding and fission of membrane vesicles from the plasma membrane, and hence, microvesicle surface proteins are largely dependent on the composition of the membrane of origin [35, 36]. It should be pointed out that because EVs are produced by virtually all cells, every viral preparation is probably a mixture of virions and EVs. In contrast to that played by exosomes, the role played MVs in viral infection is largely unknown. Further research involving a critical comparison between viral particles and different types of EVs may lead to an improved understanding of both viral pathogenesis and EV functions. Interestingly, our LC–MS/MS analysis revealed that the exosomes presented many known virus-specific receptors. The interaction of virions with these virus receptors was initially investigated with CVA16 and Zika virus, with the results suggesting that virions interacting with exosomes via specific virus receptors is a widespread mechanism of viral infection and spreading. Indeed, a recent study demonstrated that ACE2-enriched EVs enhanced the infectivity of SARS-CoV-2, making the virus much more efficient than the virus alone [37]. Hence, it is of great interest and urgency to fully understand the multifaceted role of EVs in the viral infection cycle, especially before considering EVs therapeutic options.

Remarkably, a study by Chen *et al* demonstrated that clusters of enteroviral particles were packaged within phosphatidylserine (PS) lipid-enriched vesicles of size range 250–350 nm and nonlytically secreted out of cells in the early stage of infection. Although these viral particles in vesicles were more efficient in establishing infection than free viral particles, the infection capability of these vesicles was dependent on both the virus-specific receptor of the recipient host cells and the vesicular PS lipids [38]. Our study showed that by binding with exosomes, CVB3 can be successfully transferred into receptor-negative lymphocytes to establish productive infection. It is tempting to speculate that the two types of vesicles may have different roles in viral pathogenesis or tissue tropism. Indeed, subsequent studies confirmed that the PS vesicles-containing high-weight infectious units allowed enteroviruses to increase MOI to better override host defenses while keeping genetic relatedness high, preventing invasion by defective viruses [39, 40]. Our findings showed that by binding exosomes, viruses may have a broader host range because the infection is not restricted by the presence of cellular receptors. Importantly, the entry of EVs is more promiscuous than that of viruses, and clathrin dependent, caveolae dependent, macropinocytosis, phagocytosis and lipid raft-mediated internalization have all been shown to contribute to the entry of EVs [24]. Several specific protein-protein interactions, such as tetraspanins and integrins, have also been reported. Our results demonstrated that exosomes mediated CVB3 entry through a variety of endocytic pathways, as well as tetraspanins-dependent pathways. These multiple approaches of uptake/infection provide various possibilities for viral transmission in different cell types or tissues. In addition, CVB3 virions have previously been observed within autophagosome-like vesicles in differentiated neural progenitor cells [41], suggesting the release of the vesicles harboring virions is associated with the autophagy pathway. However, we did not find CVB3 virions inside the purified exosomes in any of our experiments. This discrepancy between studies may be results of cell type specificity during CVB3 infection. CVB3 has been previously shown to induce autophagic signaling in most susceptible host cells, such as epithelial cells [42,43]. In contrast, no change in autophagic signaling has been seen in neural progenitor or stem cells following infection with CVB3, and modulation of autophagy did not alter virus titers [44]. It is likely that the presence of CVB3 virions within autophagosome-like vesicles is specific to a few specific host cells. To date, data supporting the role played CVB3 within autophagosome-like vesicles in viral tropism, spread, and pathogenesis are rare. The infectivity of encapsulated CVB3 virions need to also be further explored.

CVB3 infection not only induces a variety of acute diseases but also results in persistent infections. Viral persistence in cellular constituents of the immune system may lead to crucial biological consequences. The key obstacle to viral persistence is failure of a host immune system to eliminate the virus during acute infection [12,45,46]. Previous studies confirmed that splenic T and B lymphocytes were persistently infected by CVB3 in the absence of readily detectable CAR receptor [8,47]. Persistent infection of lymphocytes provides CVB3 virus with an important way of disrupting the immune system. For example, neutralizing antibodies are considered to be critical to the control of viral infections. It is therefore tempting to suggest that infection by CVB3 of B cells represents an attempt by the virus to block antagonistic antibody production [48–50]. Our study showed that splenic B and T cells in Rab27a-KO mice with repressed exosomes exhibited a low rate of CVB3 infection, resulting in a high level of specific neutralizing antibody production, a lower viral load in cardiac tissue and minor myocarditis pathogenesis. The infiltration of mononuclear leukocytes has previously been shown to be the characteristic hallmark of CVB3-induced acute and ongoing myocarditis [51]. Exosomes provide a permissive route for CVB3 infection of receptor-negative mononuclear cells, which may lead to viral spreading to distant sites, including the myocardium. All the results in our study clearly verified that CVB3 infection mediated by exosomes disrupts the normal function of immune cells and the homeostasis-related immunological response to antigens, resulting in persistent infection and chronic enteroviral myocarditis.

## Materials and methods

### Ethics statement

All animal experimental protocols were approved by the Soochow University Animal Care Committee and followed the ‘Guide for the Care and Use of Laboratory Animals’ published by the Chinese National Institutes of Health. The research protocols were conducted in accordance with the animal behavioral guidelines, using approved protocols from the institutional animal care committee (#202201A0129).

### Cell lines and viruses

Cell lines Caco-2, HT-29, Vero E6, RD, NIH 3T3 and 293T were cultured in Dulbecco’s Modified Eagle’s Medium (DMEM, Hyclone) containing 10% (v/v) fetal bovine serum (FBS, Hyclone) at 37°C in a 5% CO_2_ humidified incubator. HL-1, a continuously proliferating cardiomyocyte cell line, was cultured in Claycomb Medium (Sigma) with 10% FBS, 0.1 mM norepinephrine(Sigma) and 2 mM L-glutamine(Gibco). Sf9 cells were cultured in Sf-900II medium (Gibco) at 27°C containing 10% FBS(Gibco). All cell lines were obtained from American Type Culture Collection (ATCC, USA). The stock of CVB3 virus (Woodruff strain, GenBank: U57056.1) was propagated through Vero E6 cells. Viral titrations were performed with 10-fold serial dilutions in Vero E6 cells. Three days after inoculation, CPE was scored, and the Reed-Muench formula was used to calculate the TCID50 [52]. For standard plaque forming assay, NIH 3T3 cells in 12-well plates were infected with viruses for 1 h and cells were overlaid with 1% low-melting point agarose (Thermo) in DMEM containing 2% FBS. After further incubation for 3 days, the cells were fixed with 4% formaldehyde and stained with 0.2% crystal violet to visualize the plaques.

### Isolation and purification for subpopulation of extracellular vesicles and CVB3 virion

WT Cells or knockout cells were incubated with CVB3(0.05 TCID_50_) for 1h, then the cells were washed with PBS and switched to DMEM supplemented with 10% EV-depleted FBS(System Biosciences lnc) for the production of microvesicles, exosomes or virus particles. After 24 hours, cell debris and apoptotic bodies was pelleted at 2,500×g for 20 min(twice). The supernatant was collected and ultracentrifuged at 16,000× g for 45 min at 4°C to pellet microvesicles. Supernatant from the 16,000× g step was passed through a 0.22 μm pore PES filter (Millipore, USA) and next subjected to ultracentrifugation at 100,000 g for 90 min(Beckman Coulter Optima L-100 XP, Beckman Coulter). Resuspend the pellets in PBS and repeat centrifuged 90 min at 100,000 g at 4°C to remove soluble serum and secreted proteins. Then the pellet (mixture of exosomes and virion) was resuspended in PBS by pipetting. For immune-selection, the pellet in tube was incubated with CVB3 VP1 antibody (Wako Pure Chemical Industries, Ltd.) at 4°C overnight and mixed with Protein A/G Magnetic Beads (Millipore) for an additional 60 minutes. The tube placed into the magnetic stand to capture the beads resulted in collecting virion, then transferred supernatants to new centrifuge tube(purified exosomes without virion). For iodixanol density gradient ultracentrifugation, after the discontinuous gradient was formed, 1mL pellet sample in PBS(mixture of exosomes and virion) was layered on top of these gradients and centrifuged in SW40 rotor (Beckman Coulter) for 16 h at 120,000 ×g, 4°C, each individual 1 mL fraction was transferred to new tubes(F1–F10) and subjected to western blot analysis. Fraction densities were calculated from refractive index measured using a refractometer (Carl Zeiss).

### Nanoparticle-tracking analysis, Transmission and scanning electron microscope

Particle size, concentration and morphology were measured by Nanoparticle Tracking Analysis and electron microscope respectively as our previous description [53].

### Western blot

Samples of treated cells, exosomes and MVs fraction were lysed in RIPA buffer(Santa Cruz, USA) for 20 min on ice and cleared lysate was collected by centrifugation for protein separation on 10% SDS-PAGE gels(Bio-Rad, USA). The proteins were transferred onto Immobilon-FL PVDF Transfer Membrane(Millipore) and detected with appropriate primary antibodies at 4°C overnight, followed by incubation with HRP-conjugated anti-rabbit IgG or anti-mouse IgG (Southern Biotech) secondary antibodies. Membranes were exposed using an enhanced chemoluminescence(NcmECL Ultra) system(NCM Biotech, Suzhou, China) and band intensities were quantified by ImageJ software(NIH). Primary antibodies against CD63(BD Biosciences), CD9(Sigma, #SAB4503606), DAF(#31759), CVB3 VP1(Dako,#M706401-2), Annexin A1(#32934), Annexin A5(#8555), GM130(#12480), Calnexin(#2679) (all from Cell Signaling Technology, USA), CAR(#ab272711), Alix(#ab186429), SCARB2(#ab240186), Tyro3(#ab109231), GAPDH(#ab8245)(all from Abcam, USA), CAV16 VP1(GTX132338), CAV16 3AB(GTX132344), Zika Envelope(GTX133314), Zika Capsid(GTX133317)(All from GeneTex, Inc. Taiwan, China).

### Immunofluorescence staining

Immunofluorescence staining was performed according to standard procedures. For internalization assay, cells were grown to subconfluency on coverslips under DMEM complete medium containing 100μM Dynasore, 200μM EIPA, 1μM Annexin V, 5μg/mL Heparin or 10μg/mL Filipin(all drugs purchased from Sigma-Aldrich) for 1 hour, followed by treating with CVB3-GFP at 0.1 TCID_50_ or exosomes-CVB3-GFP complex(contain 0.1 TCID_50_ CVB3-GFP) for 4h. For antibodies blocking, cells were pretreated with CD9(#SAB4503606, Sigma) or CD81(#ab79559, Abcam) antibody for 2h and incubated with CVB3-GFP or exosomes-CVB3-GFP complex for 4h. After various treatments, cells were washed three times with PBS and treated with DiI (1,1’-Dioctadecyl-3,3,3’,3’-tetramethylindocarbocyanine perchlorate; Sigma-Aldrich) for 10 min at 1 μM to label the cell membrane. For colocalization experiments, CVB3-GFP-infected cells(0.05 TCID_50_) were fixed with 4% paraformaldehyde and permeabilized in 0.1% Triton X-100 and sequentially incubated with primary and Alexa Fluor 594-tagged secondary antibody. The primary antibodies used were CD63(#ab1318, Abcam), CD9(#SAB4503606, Sigma). DAPI dyes (Sigma) was used for cell nucleic acid stains. Fluorescence microscopy and images were analyzed using the Nikon Eclipse Ti confocal microscope (Tokyo, Japan).

### Agarose gel eletrophoresis

RNA of cells, exosomes or virion samples were isolated using TRIzol reagent(Life Technologies, USA) according to manufacturer’s instructions. The reverse transcription was performed using PrimeScript RT Master Mix for RT-PCR(TaKaRa). RT products used DreamTaq Green PCR Master Mix(Thermo Scientific) to amplify the CVB3 RNA. The PCR products were resolved on 1.5% agarose/TAE gels containing ethidium bromide. All PCR primer sequences were as follow:

Full-length viral genome:

Forward: 5’-TTAAAACAGCCTGTGGGTTGATCC—3’,

Reverse: 5’-CGCACCGAATGCGGAGAATTTACCCC-3’;

P1:

Forward: 5’-AGCCAATCGGCAGGATTTCA-3’,

Reverse: 5’-TCAGCTTCCGGTACACACAC-3’;

P2:

Forward: 5’-AGTGTGTGTACCGGAAGCTG-3’,

Reverse: 5’-TTGATTGTAGCCCCACGTCC-3’;

P3:

Forward: 5’-GCCAAGCGGTATGCTGAATG-3’,

Reverse: 5’-GTAACTCCGCTGGGTTGGAA-3’;

P4:

Forward: 5’-TAGACATAGCGTGGGGACCA-3’,

Reverse: 5’-ATCAAGATGGTTGGCCCAGG-3’;

P5:

Forward: 5’-GATCCACGTCTCAAGGCCAA-3’,

Reverse: 5’- CCATGGGTACGATGCGATCA-3’.

### Preparation of exosomes-CVB3 binding

Purified exosomes were incubated with free CVB3 virion or CVB3-GFP virion at approximately 1:3 ratio of numbers(0.5 mL total volume mixture in PBS). After 4h, Mixture was subjected to CD9 antibody(#ab236630, Abcam) followed by Protein A/G Magnetic Beads (Millipore)with 25μl for 2h. Mixture placed into the magnetic stand to capture the immune complex beads and washed with cold PBS five times. Magnetic beads resuspended in 100μl Release Buffer(20mM trietholamine, 2M NaCl, pH = 6.0) in tubes and incubated for 1h at room temperature while shaking at 800 rpm to release the magnetic beads. Placed the tubes on magnetic stand to collect the supernatant with the released exosomes-CVB3 complex and ready for Nanoparticle-tracking analysis.

### Real-time PCR analysis and determination of viral RNA copy number

Total RNA was isolated from treated cells or mouse tissues using TRIzol reagent according to the manufacturer’s specification and then reverse transcribed using random hexamers with the reverse-transcription kit (TaKaRa). The cDNA was subjected to quantitative PCR using SYBR-Green Master Mix (Life Technologies) on an ABI QuantStudio 6 F​lex for 40 cycles. The sequences of inflammatory cytokines primer pairs were as follow: IL-1β: Forward:5’-TCACAGCAGCACATCAACAA-3’, Reverse: 5’-TGTCCTCATCCTGGAAGGT-3’; TNF-α: Forward:5’- AAGCCTGTAGCCCACGTCGTA-3’, Reverse: 5’- GGCACCACTAGTTGGTTGTCTTTG-3’; IL-6: Forward:5’- ACAACCACGGCCTTCCCTACTT -3’, Reverse: 5’- CACGATTTCCCAGAGAACATGTG-3’. qRT-PCR was used to determine the viral RNA copy number. The primers were CVB3-F (5’-AACGCCAAAACAACGGATGG-3’) and CVB3-R (5’- GATCTGGGTCTGGGGGTAGT-3’). A fragment corresponding to nucleotides 4035–4529 of CVB3 Woodruff strain was adjusted to a concentration gradient and was used as a standard to calculate the copy number of viral RNA. The reaction condition was as follows: 95°C for 10 min, followed by 40 cycles consisting 95°C for 15 s, and 60°C for 1 min.

### Construction of stable-expression cells

The lentiviral expression vector pLVX-shRNA(for knockdown, Clontech)or pLVX-IRES (for overexpression, Clontech)was used to generate lentivirus particles. containing specific sequences for human CAR or DAF(sequence synthesis by GenePharma Inc, Shanghai, China). The lentiviral packaging plasmid pMD2.G (AddGene 12259) and psPAX2 (AddGene 12260) were obtained from AddGene. HEK-293T cells were transfected with 10μg pLVX-shRNA/pLVX-IRES, 5μg psPAX2, and 3μg pMD2.G using the jetPRIME(Polyplus-transfection, France) according to the manufacturer’s instructions to package virus. Harvest the lentiviral supernatants and filter through 0.45-μm filter to remove cellular debris. 293T cells were then infected with lentivirus particles at MOI = 2 for three days. After limiting dilution, single clones were selected in the presence of puromycin (2μg/mL,Sigma) for three weeks and assayed for expression of the RNA by PCR or protein by western blot.

### Generation of knockout cell lines

The lentiviral vector lentiCRISPRv2, which expresses clustered regularly interspaced short palindromic repeats (CRISPR/Cas9) and guide RNA (gRNA) (AddGene 73179), the lentiviral packaging plasmid used pMD2.G and psPAX2 vector. Using the CRISPR online design tool (http://www.genome-engineering.org/crispr/), we generated the guide RNAs (sgRNAs) targeting the human TSG101, Alix or Rab27a gene were subcloned into the lentiCRISPRv2 vector. HEK-293T cells were transfected with those vectors to package lentivirus. Caco-2 cells were then infected with lentivirus and then single clones were selected in the presence of puromycin (2μg/mL). Knockout cells were assayed for expression of the proteins by western blot.

### The tandem mass spectrometry (LC-MS/MS)

Purified EVs were isolated from different cells as described above. The LC-MS/MS experiments were performed by Novogene Bioinformatics Technology Co. Ltd (Beijing). Raw data files were searched against the Uniprot_Human protein database. Peptide and scan-counting was performed assuming as positive events those with a FDR equal to or lower than 5%.

### Animal experiments

For detection of the copy number of CVB3 RNA in mouse organ tissues, specific pathogen-free male BALB/c mice aged six weeks were purchased from the GemPharmatech Co. Ltd. (Nanjing, China). The mice were orally inoculated after 10 h of fasting at 5×10^6^ TCID_50_ per mouse for 24h. Before samples were collected, all the mice were injected with anesthetics and then perfused transcardially with 50 ml of cold PBS per mouse. The organs were vacuum-dried, weight and frozen instantly in liquid nitrogen. Total RNA was extracted from individual tissues using TRIzol(Life Technologies, USA) and quantified using a spectrophotometer.

For exosomes-mediated infection, purified exosomes from CAR-KD or WT cells were incubated with CVB3-GFP virion at approximately 1:3 ratio of numbers. Mixture was subjected to CD9 immune-selection and then released the beads to collect exosomes-CVB3-GFP complex. Equal CVB3 RNA copy number of exosomes-CVB3-GFP were injected into the tail veins of BALB/c mice (1×10^12^ copy number per mouse, n = 5) to ensure a comparable viral inoculum. Mice tissues were then fixed in 4% paraformaldehyde overnight and embedded in paraffin. Then paraffin samples were cut into 5μm tissue sections, de-waxed in xylene for 5min (three times) and rehydrated with alcohols in decreasing concentration (100%, 90%, 80%, 70%, 60%) at room temperature. After washing in PBS, tissue sections were stained with DAPI (Beyotime Biotechnology, Shanghai, China).

For CVB3 infected experiments, the Rab27a knockout mice(C57BL/6J background) were constructed by Cyagen Biosciences (Suzhou, China). The mice were bred and maintained in specific pathogen free (SPF) environment at the Laboratory Animal Center of Soochow University.

The homozygous Rab27a-KO mouse was identified by PCR using the primer: Forward primer: 5’-CATCTCCCTGGTCTCTATAAAATC-3’, Reverse primer: 5’-ACATCCATAAAACATATTCCCCTC-3’(one band at 476bp). Six weeks-old of Rab27a-KO mice or wild-type mice were orally inoculated after 10 h of fasting at 5×10^6^ TCID_50_ per mouse. The infected mice were continuously observed to record body weight and death. Mice were dissected at 1, 3, 5, 7, 9, 11, 14, 28 and 42 day to collect cardiac tissue, spleen and blood to screen virus replication, inflammatory cytokine and antibody titer. All animal experiments were performed in accordance with the recommendations of the Guide for the Care and Use of Medical Laboratory Animals (Ministry of Health, PR China, 1998) and the guidelines of the Laboratory Animal Ethical Commission of Soochow University(#202201A0129).

### Preparation and purification of murine lymphocytes

After aseptically extraction, the spleens were gently pressed through cell strainers (70 μm) with the plunger of a syringe into PBS in a Petri dish, and the suspensions were treated with RBC lysis buffer on ice for 10 min and washed with PBS, cell pellets were resuspended in cold buffer (2 mM EDTA/PBS; 0.5% BSA). Thereafter, splenocytes were prepared for magnetic-activated cell separation (STEMCELL Technologies) by incubation with magnetic beads conjugated with CD3(#19851RF, STEMCELL) or CD19(#19854RF, STEMCELL) antibodies. Carefully pipetted the enriched cell suspension into new tubes and used for RNA isolation or western blot.

### ELISA for CVB3 neutralizing antibody

For CVB3 specific neutralizing antibody assays, 96-well polystyrene high-binding microplates (Corning, USA) were coated with 0.1μg/100μl recombinant CVB3 VP1 protein(Sino Biological, China) in carbonate-bicarbonate buffer pH 9.6, and the plates were incubated at 4°C overnight. The tested sera were diluted at 1:100 and added to each well, and four multiple wells were set for each sample, and then incubated at 37 °C for 60 min, followed by goat anti-mouse secondary antibodies conjugated with HRP (Southern Biotech,1:5000 dilution), and incubated at room temperature for 45 min. The reaction was developed by TMB substrate and the optical densities(OD) at 450 nm were determined.

### ELISpot

SARS-CoV-2-specific cellular immune responses in ferrets were assessed by a Murine IFN-γ ELISpot Kit (#ab64029, Abcam) following the manufacturer’s instructions. In brief, 1×10^5^ of peripheral blood mononuclear cells (PBMCs) of infected mice were produced by density gradient sedimentation and stimulated with inactivated CVB3 in a pre-coated ELISpot plate for 12 h in a 37°C humidified incubator with 5% CO_2_. The plate was washed five times with PBST and sequential incubation with biotinylated detection antibody, streptavidin-AP and developed with BCIP/NBT buffer. Plate was washed extensively in deionized water to stop colour development and dried in the dark, and the spots were counted in ImmunoSpot Series 3 Analyzer (Cellular Technology). The result was expressed as the number of CVB3 specific spots per 1 million PBMCs.

### Echocardiography

Anesthesia and echocardiography were acquired as previously described [54].

### Statistical analysis

Data were shown as means ± SD. Statistical analysis of the data was performed using the GraphPad Prism (Version 8.0) statistical program. The unpaired Student’s t-test was used to compare differences between two groups, whereas comparison of multiple groups was performed using ANOVA with post hoc tests to compare differences between individual groups. P-values were either listed or represented by the following number of asterisks: * p < 0.05, ** p < 0.01, and *** p < 0.001.

## Supporting information

S1 Fig Exosome coupling with CVB3 virions depended on CAR but not DAF. (A, B and C) Proteomic analysis of exosomes samples by LC-MS/MS. The purified exosomes were isolated from three different-type cell lines(Caco-2, A549 and THP-1 cells). Venn diagram describing the relationships between exosomes and known virus-receptors that searched against UniProt Knowledgebase(UniProtKB). Numbers in box represent the quantity of the common proteins. (D, E and F) Cells were infected with coxsackievirus A16(CVA16) or Zika virus at 0.1 TCID_50_, then the cells were washed and switched to medium supplemented with EV-depleted FBS for the production of exosomes and virus particles for 24h. The supernatants were performed by differential ultracentrifugation according to description in Fig 1, followed by immuno-selection with anti-CD9. The expression levels of viral proteins, viral receptors and exosomal marker CD9 and Alix in different pellet were measured by western blot analysis(E and F). The loading amount of each sample was 100μg total protein.
(TIF)

S2 Fig Exosomes carrying CVB3 exhibited highly efficient infection of receptor-negative cells through various entry routes. (A, B and C) Purified exosomes derived from WT cells, CAR^KD^ Caco-2 cells or DAF^KD^ Caco-2 cells were incubated with CVB3-GFP virion for 4h. Then each group of exosomes-CVB3-GFP complex was treated 293T cells for different time-point(A). Fluorescence microscopy(B) and flow cytometry(C) analysis of GFP-positive cells proportion. PBS treatment as the negative control. All the data are shown as mean ± SD of three independent experiments. (*p<0.05, **p<0.01, ns: no significance).
(TIF)
