# Supplementary figures and images for: Exosomes mediate Coxsackievirus B3 transmission and expand the viral tropism
Source: PLoS Pathog. 2023 Jan 12;19(1):e1011090. doi: 10.1371/journal.ppat.1011090 (PMC9888687; doi:10.1371/journal.ppat.1011090)

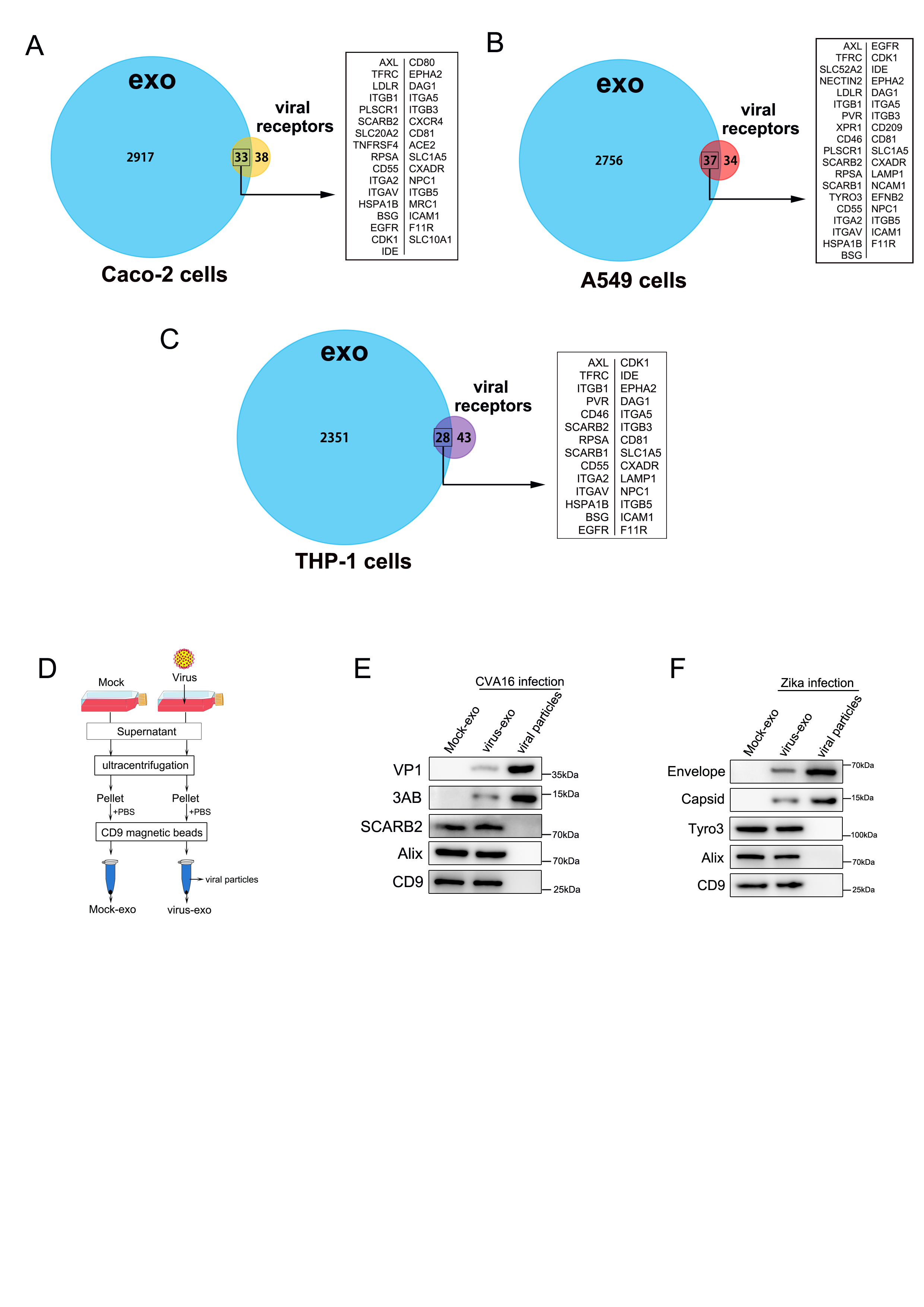

Supplement: S1 Fig — (A, B and C) Proteomic analysis of exosomes samples by LC-MS/MS. The purified exosomes were isolated from three different-type cell lines(Caco-2, A549 and THP-1 cells). Venn diagram describing the relationships between exosomes and known virus-receptors that searched against UniProt Knowledgebase(UniProtKB). Numbers in box represent the quantity of the common proteins. (D, E and F) Cells were infected with coxsackievirus A16(CVA16) or Zika virus at 0.1 TCID50, then the cells were washed and switched to medium supplemented with EV-depleted FBS for the production of exosomes and virus particles for 24h. The supernatants were performed by differential ultracentrifugation according to description in Fig 1, followed by immuno-selection with anti-CD9. The expression levels of viral proteins, viral receptors and exosomal marker CD9 and Alix in different pellet were measured by western blot analysis(E and F). The loading amount of each sample was 100μg total protein. (TIF) [file ppat.1011090.s001.tif]

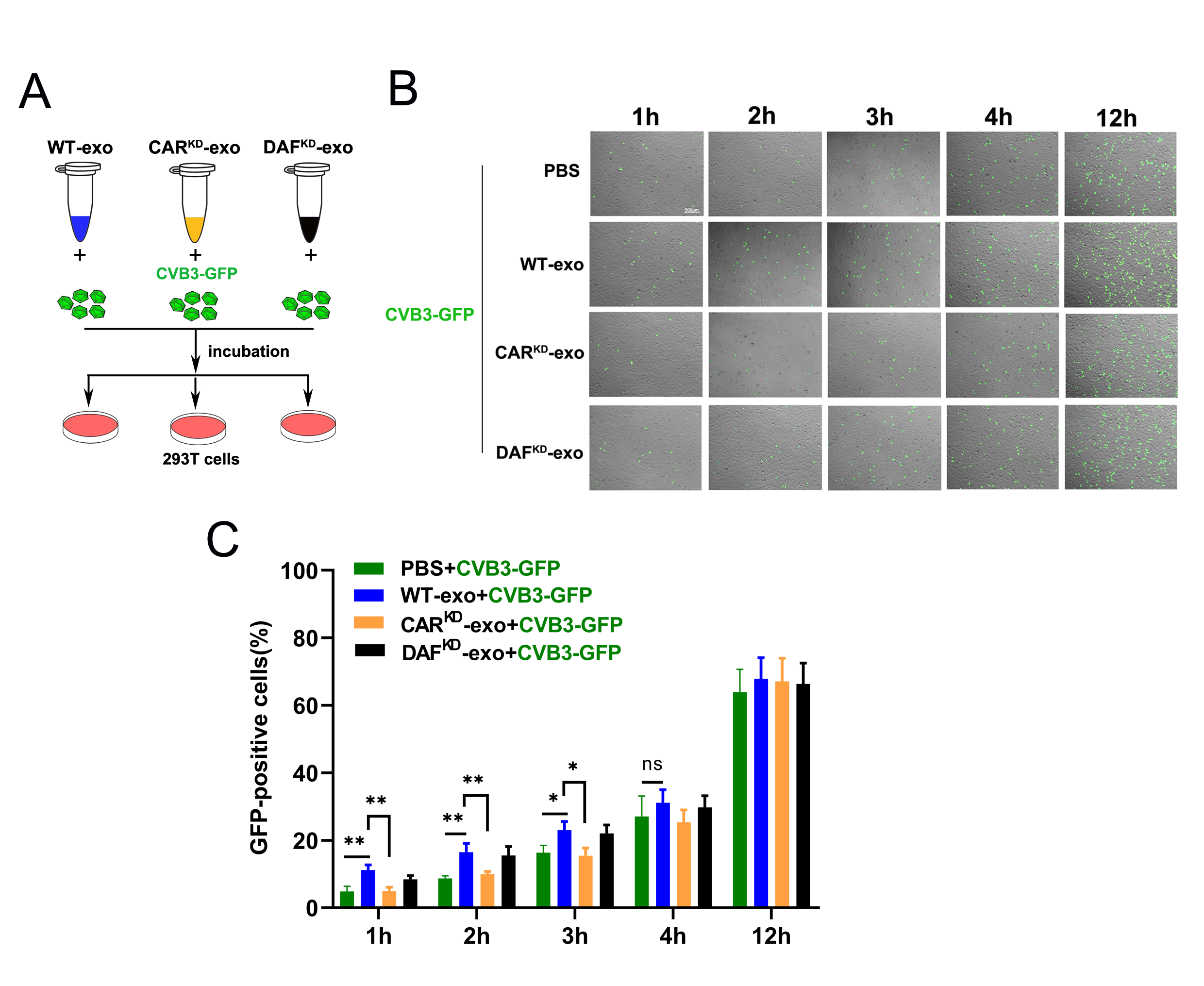

Supplement: S2 Fig — (A, B and C) Purified exosomes derived from WT cells, CARKD Caco-2 cells or DAFKD Caco-2 cells were incubated with CVB3-GFP virion for 4h. Then each group of exosomes-CVB3-GFP complex was treated 293T cells for different time-point(A). Fluorescence microscopy(B) and flow cytometry(C) analysis of GFP-positive cells proportion. PBS treatment as the negative control. All the data are shown as mean ± SD of three independent experiments. (*p<0.05, **p<0.01, ns: no significance). (TIF) [file ppat.1011090.s002.tif]
